# Titin-dependent biomechanical feedback tailors sarcomeres to specialized muscle functions in insects

**DOI:** 10.1126/sciadv.ads8716

**Published:** 2025-05-09

**Authors:** Vincent Loreau, Wouter H. Koolhaas, Eunice HoYee Chan, Paul De Boissier, Nicolas Brouilly, Sabina Avosani, Aditya Sane, Christophe Pitaval, Stefanie Reiter, Nuno Miguel Luis, Pierre Mangeol, Anne C. von Philipsborn, Jean-François Rupprecht, Dirk Görlich, Bianca H. Habermann, Frank Schnorrer

**Affiliations:** ^1^ Aix Marseille University, CNRS, IBDM, Turing Centre for Living Systems, Marseille, France.; ^2^ Max Planck Institute of Biochemistry, Martinsried, Germany.; ^3^ Department of Neuroscience and Movement Science, Medicine Section, University of Fribourg, Fribourg, Switzerland.; ^4^ Max Planck Institute for Multidisciplinary Sciences, Göttingen, Germany.; ^5^ Aix Marseille University, CNRS, CPT, Turing Centre for Living Systems, Marseille, France.

## Abstract

Sarcomeres are the universal contractile units of muscles that enable animals to move. Insect muscles display a remarkable functional diversity: they operate at extremely different contraction frequencies (ranging from ~1 to 1000 hertz) and amplitudes during flying, walking, and crawling. This is puzzling because sarcomeres are built from essentially the same actin-myosin components. Here, we address how functionally different sarcomeres are made. We show that the giant protein titin and the regulation of developmental contractility are key for the sarcomere specializations. I-band titin spans and determines the length of the sarcomeric I-band in a muscle type–specific manner. Unexpectedly, I-band titin also rules the length of the force-generating myosin filament using a feedback mechanism that is modulated by myosin contractility. We propose a model of how sarcomere specializations in insects are tuned, provide evidence for this model, and discuss its validity beyond insects.

## Teaser

A mechanical mechanism instructs sarcomeres to fulfill the specific needs of different muscle types.

## INTRODUCTION

Sarcomeres are the basic contractile units of striated muscles across the animal kingdom. They display a stereotypic architecture: Regularly arrayed actin filaments are anchored with their plus ends at the Z-discs, central bipolar myosin filaments are linked at the M-band, and both filaments are connected by large titin molecules (*1*–*4*). The myosin part of the sarcomere, called the A-band, maintains its length during sarcomere contraction, while the myosin-free zone, called the I-band, shortens during contraction (*5*). Despite this conserved architecture, muscle function—as determined by its specific contraction frequency, dimension, and force production—varies markedly across animal evolution. Mammalian muscles generally contract with low hertz frequencies, shorten about 30% of their length, and produce an average power of 5 to 15 W/kg for the heart or 20 to 30 W/kg for skeletal muscles during continuous blood pumping or endurance movements, respectively (*5*–*7*). In contrast, invertebrate muscles achieve a much wider range of contraction regimes: insect flight muscles contract with hundreds of hertz [up to 1000 Hz in small midges (*8*)], while only shortening 1 to 2% to produce a mechanical power of more than 80 W/kg to achieve long-range flight (*9*–*11*). In the same insect, leg muscles sarcomeres contract with low hertz and shorten up to 50% to power walking or mating. At the extreme end of the evolutionary specialization are the mandible muscles of leafcutter ants, which produce the highest forces per body mass (>25,000 N/kg body weight to cut leaves) with specialized long sarcomeres (*12*, *13*) and the gut muscles from annelids (Lophotrochozoans) with a sarcomere length of up to 40 μm (*14*). How can evolution tune the muscle parameters to these extremes in a reproducible manner while maintaining the basic periodic architecture of the contractile sarcomeres?

In mammalian muscles, titin acts as the blueprint for sarcomere architecture: The titin N terminus is bound to α-actinin at the Z-disc, and its C terminus is embedded at the M-band in the middle of the sarcomere. This led to the titin ruler hypothesis: Sarcomere length is two times the length of titin, with titin also controlling I-band and A-band lengths (*2*, *3*, *15*–*17*). In the I-band region, titin contains long proline, glutamic acid, valine and lysine (PEVK)-rich sequences, which act as molecular springs and change length during the sarcomere contraction-relaxation cycles, depending on the pulling forces on the titin protein. These forces are called “passive forces” and are produced by the antagonist muscle that stretches the relaxing muscle (*3*, *18*, *19*). Alternative splicing of titin’s I-band spring region, which removes its PEVK-rich sequence only in the heart, results in 2-μm-long sarcomeres with shorter I-bands in the heart and 3-μm-long sarcomeres with longer I-bands in human skeletal muscles (*3*, *20*–*22*). In both types, the myosin filament length (the A-band length) is fixed to 1.6 μm by the constant A-band part of the titin molecule (*16*). These fixed dimensions of mammalian sarcomeres likely limit muscle specializations. In invertebrates, sarcomere functions vary much more markedly; however, the mechanisms underlying these morphological and mechanical sarcomere specializations are not yet known.

## RESULTS

### A titin evolutionary tree

To better understand how insects can build sarcomeres with vastly different functional properties from similar components, we used *Drosophila* as a genetically tractable model. We focused on two representative muscle types; first, the larval muscles, which house 8-μm–long relaxed sarcomeres with only a small (less than 1 μm) actomyosin overlap, and second, the indirect flight muscles, which contain 3.4-μm–long sarcomeres with more than 95% actomyosin overlap (*23*, *24*). These specializations coincide with an alternative splicing of the I-band titin homolog Sallimus (Sls): Short Sls correlates with short (200 nm) I-bands in flight muscles, and long Sls correlates with long (5 μm) I-bands in relaxed larval muscles (*23*, *25*, *26*). The second titin homolog, Projectin, is located selectively at the A-band, which also varies in length between flight and larval muscles (*23*, *25*). Thus, we first investigated whether the presence of two titin homologs in invertebrates instead of one in mammals may explain the variety of muscle specializations in invertebrates.

**Fig. 1.**
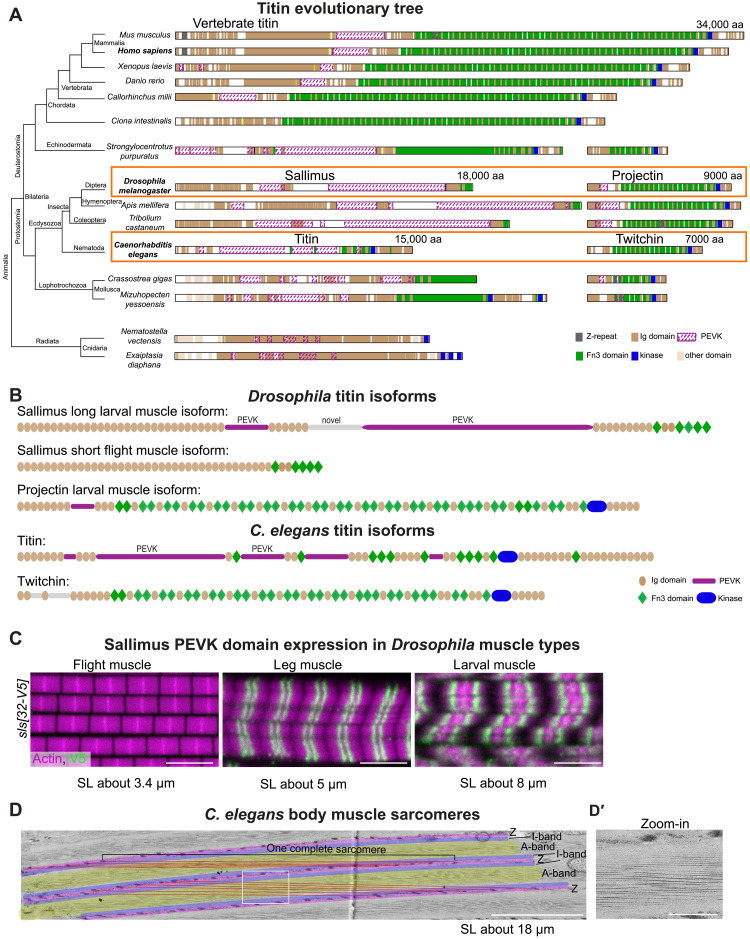
Evolution of titin homologs in animals. (**A**) Left: Evolutionary tree representing animals with striated or non-striated muscles. Right: Representative titin homologs (longest isoforms) are drawn to scale, with highlighted Z-repeats (gray), Ig domains (brown), PEVK (dashed magenta), Fn3 domains (green), and kinase domain (blue). All chordates have a long titin protein with I-band and A-band features, whereas Ecdysozoa species have two titin homologs separating I-band and A-band features into two proteins. The sizes of the mouse titin, the two *Drosophila* titins (Sallimus and Projectin), and the two *C. elegans* titins (Titin and Twitchin) are indicated. The latter four are highlighted by orange boxes. For organisms with recent genome duplications only one titin is shown (*Danio rerio*, ttn2, XP_021334745.1; *Xenopus laevis* titin-like isoform X10, XP_018091448.1; see table S1). (**B**) Domain scheme of *Drosophila* and *C. elegans* titin isoforms: Sls (short and long), Projectin, *C. elegans* Titin, and Twitchin. PEVK-rich regions are highlighted in magenta. (**C**) Flight, leg, and larval muscles of *sls[32-V5]* stained for actin (magenta) and V5 (green). Scale bars, 5 μm. (**D**) Electron microscopy image of a sectioned adult *C. elegans* body muscle, cut in the plane of a sarcomere. For better orientation, Z-disc equivalents, called dense bodies, are overlayed in magenta, I-bands in purple and A-bands in yellow. Entire sarcomeres from one dense body to the next were labeled with red lines. (**D′**) Zoom-in of indicated white square in (D). Scale bars, 5 μm (D) and 1 μm (D′).

To do so, we generated an evolutionary tree of all titin-like genes from humans to jellyfish, extending on previous studies (Fig. 1) (*27*). As expected, we found one long titin homolog in all chordates (not taking into account recent genome duplications in fish or frogs), which contains the well-known features, including a series of immunoglobulin (Ig) domains and flexible PEVK-rich parts in its I-band part, as well as Ig-Fn3 domain super-repeats in its A-band part, with a kinase domain toward the C-terminal end (Fig. 1A). All Ecdysozoa, including insects and nematodes, contain two distinct titin homologs. One I-band titin, called Sls in *Drosophila* and “Titin homolog” in *Caenorhabditis elegans*, with the typical Ig and PEVK-rich regions, and one A-band titin, called Projectin (*bt*) in *Drosophila* and Twitchin in *C. elegans*, consisting of the Ig-Fn3 super-repeats and a C-terminal kinase domain (Fig. 1, A and B). Lophotrochozoans, including mollusks and annelids, also contain two titins; however in these, the I-band and A-band features appear more mixed (Fig. 1A). One potential titin precursor is also found in jellyfish genomes (Fig. 1A). Thus, based on currently available data, the genomes in the largest part of the animal tree contain two titin homologs, which seem to share the functions of the one mammalian titin.

### Long fly sarcomeres express long titins

To investigate the function of *Drosophila* titins, we focused on the I-band titin Sls, with the rationale that *sls* mRNAs are alternatively spliced, displaying large length differences, while the alternative splicing of Projectin is only minor (*24*). The exons coding for spring-like PEVK-rich sequences are spliced out in flight muscle *sls* isoforms, while they are partially retained in leg muscles and fully retained in larval muscle *sls* isoforms (fig. S1A). Hence, the predicted proteins do contain large PEVK parts in the larval isoform but not in the flight muscle isoform (Fig. 1B). To confirm these mRNA data, we inserted V5 tags into the exons 31 or 32 of the endogenous *sls* gene (see the Materials and Methods), which code for large parts of the Sls PEVK-rich sequence, generating *sls[31-V5]* and *sls[32-V5]* (figs. S1B and S2). Staining the different muscle types indeed confirmed that the short indirect flight muscle sarcomeres (about 3.4 μm) do not express these *sls* PEVK exons, whereas leg muscle sarcomeres (about 5 μm) and long larval muscle sarcomeres (about 8 μm) do (Fig. 1C and fig. S3A). This demonstrates that sarcomere length and also I-band length [see (*23*, *25*)] indeed correlate with the presence of the flexible spring domains of *Drosophila* I-band titin.

### I-band Titin length affects sarcomere length

We next asked if the presence of the spring domains in Sls is instructive for sarcomere length and I-band length, as was shown for mammalian titin (*21*). To answer this question, we used the fact that our *sls[31-V5]* and *sls[32-V5]* not only inserted a V5-tag but replaced the entire PEVK exon 31 or 32 with the V5-tag. In addition, we deleted exon 31 or 32 entirely by replacing them individually with an FRT site, resulting in *sls[31-FRT]* and *sls[32-FRT]*. Last, we used *sls[31-FRT]* and *sls[32-FRT]* to generate *sls[*∆*31-32]*, a *sls* allele with both PEVK exons deleted (figs. S1B and S2). The CRISPR-generated *sls[31-dsRed]* and *sls[32-dsRed]*, which contain a transcriptional STOP and thus likely result in truncated Sls proteins, show the expected early larval lethality with severe sarcomere phenotypes (fig. S3, B and C). All other PEVK *sls* deletion alleles were homozygous viable and able to fly, showing that these Sls proteins are functional (fig. S3B).

**Fig. 2.**
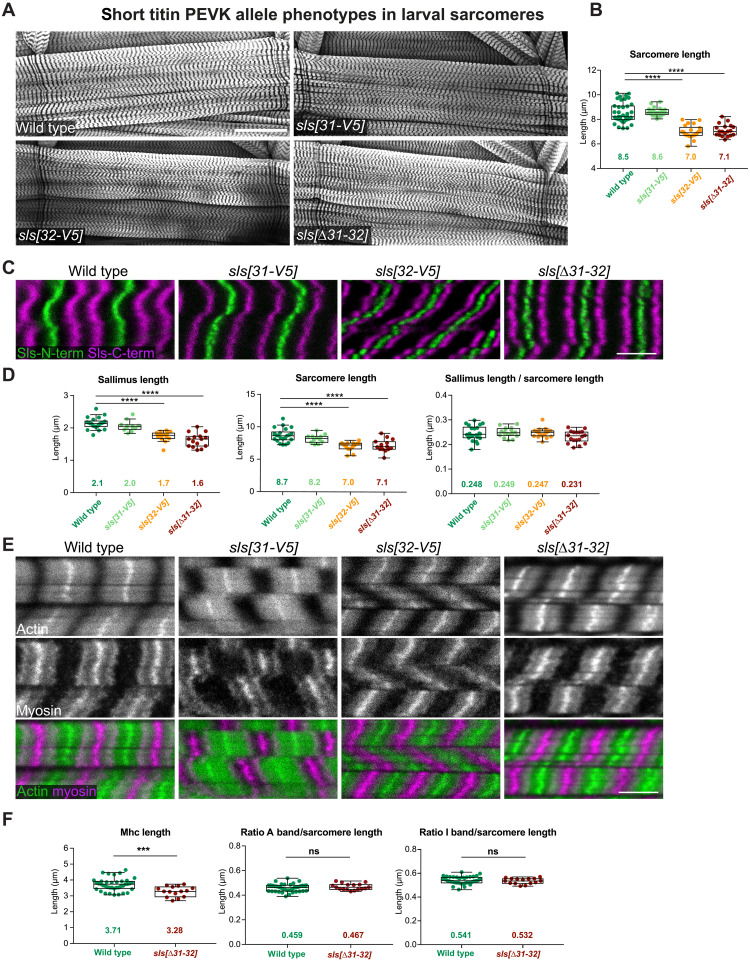
Reducing long titin PEVK length causes shorter larval sarcomeres. (**A** and **B**) Wild-type (*w[1118]*, *N* = 39), *sls[31-V5]* (*N* = 21), *sls[32-V5]* (*N* = 22), and *sls[*∆*31-32]* (*N* = 19) third instar larval VL3 muscles stained for actin (phalloidin). Scale bar, 100 μm. (B) Quantification of sarcomere length from muscle images as shown in (A). Note the shorter sarcomeres in *sls[32-V5]* and *sls[∆31-32]*. Tukey’s multiple comparisons test, ****P < 0.0001. Plots are Tukey’s box and whisker plots with median as lines, first quartile and second quartile as box, and whisker size is 1.5 times the interquartile range (IQR). All animals measured are represented as dots and average values are noted as numbers. This applies for all figures. (**C** and **D**) Wild-type (*w[1118]*, *N* = 22), *sls[31-V5]* (*N* = 12), *sls[32-V5]* (*N* = 23), and *sls[∆31-32]* (*N* = 18) third instar larval VL3 muscles stained with N- and C-terminal anti-Sls nanobodies (Sls-Nano2 in green and Sls-Nano42 in magenta). Scale bar, 5 μm. (D) Quantification of Sallimus length, sarcomere length, and ratio Sls/sarcomere length from muscle images as shown in (C). Tukey’s multiple comparisons test, ****P < 0.0001. (**E** and **F**) Wild-type (*w[1118]*, *N* = 39), *sls[31-V5]; sls[32-V5]*, and *sls[∆31-32]* (*N* = 17) third instar larval VL3 muscles stained for actin (phalloidin, green) and myosin (anti-Mhc, magenta). Scale bar, 5 μm. (F) Quantification of myosin filament length (A-band length), ratio A-band/sarcomere length and I-band/sarcomere length from muscle images as shown in (E). Mann-Whitney test, ***P < 0.001 and ns (not significant): *P* > 0.05.

To investigate sarcomere morphology in the shortened PEVK spring *sls* alleles, we stained relaxed larval muscles with phalloidin to visualize the actin filaments (Fig. 2A). Because we found a correlation between larval size and sarcomere length, we only analyzed L3 larval ventral-longitudinal muscles with a length between 400 and 500 μm (fig. S3D and see the Materials and Methods). Quantifying relaxed sarcomere length showed a length of 8.5 μm in wild-type larval muscles, which is not changed in *sls[31-V5]*. However, sarcomere length in *sls[32-V5]* and *sls[*∆*31-32]* muscles is reduced by about 1.5 μm (Fig. 2, A and B). The reduced length is compensated by a larger number of sarcomeres in these mutants. This demonstrates that reducing the PEVK spring in Sls does indeed result in shorter sarcomeres.

To directly measure Sls length, we stained larval muscles with a nanobody recognizing a domain close to the Sls N terminus and one close to its C terminus (*23*). We found that Sls in relaxed wild-type larval muscles is about 2.1 μm long, which does not change significantly in *sls[31-V5]*, demonstrating that the PEVK part encoded in exon 31 does not significantly contribute to Sls length in relaxed larval sarcomeres (Fig. 2D). However, Sls length in *sls[32-V5]* and *sls[*∆*31-32]* muscles is reduced to about 1.6 μm (Fig. 2D). This reduction of 0.5 μm fits approximately the length of the PEVK domain coded in exon 32 (fig. S3E). These results are very unexpected: Following the titin ruler hypothesis (*21*), these PEVK deletions should reduce the sarcomere length by subtracting twice 0.5 μm, so 1 μm, compared to wild type. However, the sarcomere length is reduced by 1.5 μm in *sls[32-V5]* and *sls[∆31-32]*. We found that the ratio between Sls protein length (which is the I-band length) and sarcomere length scales the same in wild-type and *sls* PEVK mutant sarcomeres (Fig. 2D). Together, these data show that Sls length does indeed control the I-band length of *Drosophila* larval sarcomeres; however, the length regulation of the entire sarcomere appears to be more complex.

### Titin elasticity controls myosin filament length

To determine the length of the second key filament of sarcomeres, the myosin filament, we stained larval muscles of wild type and *sls* PEVK deletions with a myosin antibody (Fig. 2E) and quantified myosin filament length with an automated tool, PatternJ (*28*) (see the Materials and Methods). We found that myosin filament length was reduced by about 0.5 μm in *sls[*∆*31-32]* compared to wild type (Fig. 2F). We confirmed this result by crossing the live myosin filament marker Myofilin-GFP into the *sls[∆31-32]* background and found a similar difference compared to wild type (fig. S3F). Thus, not only the I-band length scales with sarcomere length but also the A-band length, as the ratio between the A-band and sarcomere length stays constant in wild type and *sls[∆31-32]* (Fig. 2F). These data were unexpected, as they show that titin’s PEVK-dependent elasticity in the I-band also controls the A-band and hence myosin filament length. This is an interesting finding because it demonstrates that titin length scales sarcomere size in insects, hinting at a leverage mechanism that allows for much larger variations than the protein length itself.

### Elongating titin spring results in muscle type–specific effects

**Fig. 3.**
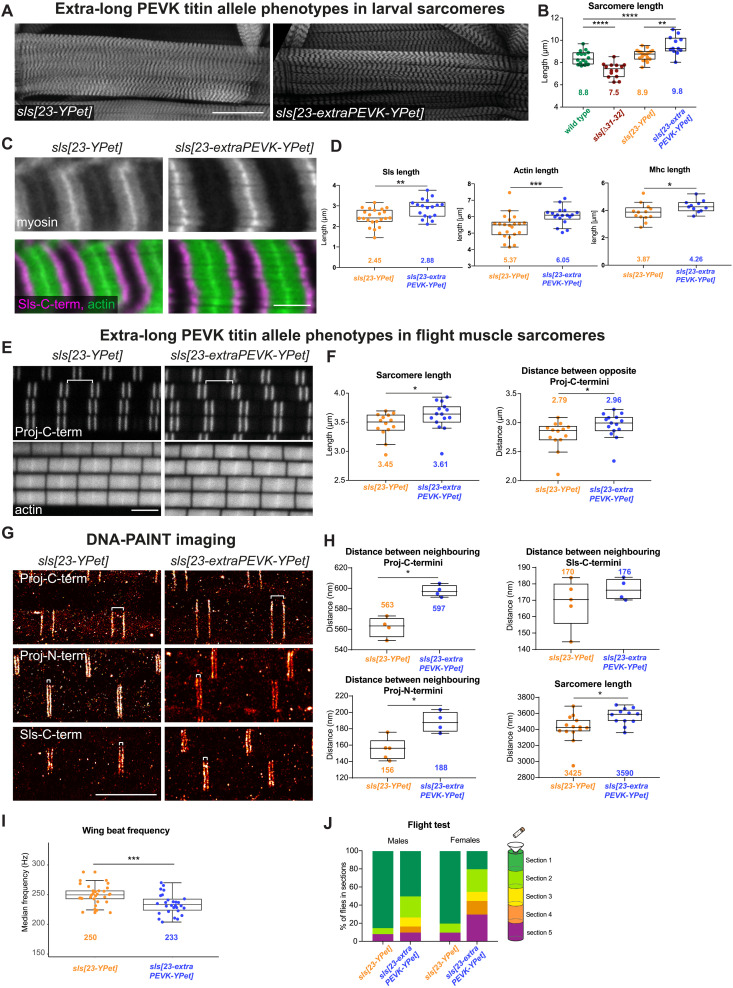
Extra titin PEVK elongates thin and thick filaments in a muscle type-specific way. (**A**) *sls[23-YPet]* and *sls[23-extraPEVK-YPet]* third instar larval VL1 muscles stained for actin. Scale bar, 100 μm. (**B**) Quantification of sarcomere length of wild type (*w[1118]*, *N* = 17), *sls[∆31–32]* (*N* = 13), *sls[23-YPet]* (*N* = 18), and *sls[23-extraPEVK-YPet]* (*N* = 14). (**C** and **D**) *sls[23-YPet]* (*N* = 23 for Sls, 13 for actin and 22 for myosin length) and *sls[23-extraPEVK-YPet]* (*N* = 18, 11, and 18) VL1 muscles stained for myosin, actin (green), and Sls-C-terminal nanobody (Sls-Nano48, magenta). Scale bar, 5 μm. Mann-Whitney test, *P < 0.05, **P < 0.01, and ***P < 0.001. (D) Quantification of Sls, actin, and myosin filament length from images as shown in (C). (**E** and **F**) Flight muscles of *sls[23-YPet]* (*N* = 14) and *sls[23-extraPEVK-YPet]* (*N* = 15) stained with C-terminal anti-Projectin nanobody (Proj-Nano37) and actin (phalloidin). Scale bar, 3 μm. (F) Quantification of sarcomere length and distance between Proj-C-termini [shown by white bracket in (E)]. Mann-Whitney test, *P < 0.05. (**G** and **H**) DNA PAINT imaging of *sls[23-YPet]* (*N* = 14) and *sls[23-extraPEVK-YPet]* (*N* = 12) stained with either anti-Projectin nanobodies [N- or C terminus, Proj-Nano29 (*N* = 5 and 4) and Proj-Nano37 (*N* = 4 for both), respectively] or anti-Sls nanobodies (C terminus, Sls-Nano39, *N* = 5 and 4). (H) Histogram of distances between bands centered around Z-discs. Mann-Whitney test, *P < 0.05. (**I**) Wing beat frequency quantification of tethered adult *sls[23-YPet]* and *sls[23-extraPEVK-YPet]* flies (*N* = 30 for each). Mann-Whitney test, ***P < 0.001. (**J**) Adult *sls[23-YPet]* and *sls[23-extraPEVK-YPet]* flies tested for flight capability (*N* = 40 for females and *N* = 60 for males). Chi-square test, *P* < 0.001.

To further explore if changing the length of I-band titin feeds back on the length of the A-band in *Drosophila* muscles, we constructed a fly strain expressing an artificially long Sls protein. To achieve this, we inserted about 4 kb of the PEVK-rich *sls* exon 32, whose deletion caused the above-shown reduction in I-band length, into exon 23 that is present in all *sls* isoforms, resulting in *sls[23-extraPEVK-YPet]* (fig. S4 and see the Materials and Methods). As a control, we only inserted a YPet marker. Adding the extra PEVK spring to Sls results in a further elongation of the already long relaxed larval sarcomeres by about 1 μm (Fig. 3, A and B), which is in line with the above-reported reduction in larval sarcomere length in *sls[*∆*31-32]*. This is accompanied by the expected gain of Sls length in *sls[23-extraPEVK-YPet]* of about 0.4 μm. In the longer *sls[23-extraPEVK-YPet]* sarcomeres, the myosin filament length does increase as well (Fig. 3, C and D). Hence, even long sarcomeres can be further elongated when lengthening the I-band ruler and in turn the A-band scales with it.

Because wild-type flight muscles do not express any Sls PEVK sequences (see Fig. 1C and fig. S3A), we predicted that the flight muscle sarcomeres of *sls[23-extraPEVK-YPet]* should also be longer. However, we were surprised to find only a very small sarcomere length increase from about 3.45 μm in control to 3.61 μm in *sls[23-extraPEVK-YPet]* flight muscles (Fig. 3, E and F), in contrast to the 1 μm increase in larval muscles. Even more unexpected is that this moderate increase is largely due to an increase in myosin filament length, which we assessed by measuring the distance between two opposite Projectin C-termini (Fig. 3, E and F) that label the myosin filament about 250 nm before its ends and hence can be resolved by confocal microscopy (*25*).

To directly measure the I-band length with super-resolution microscopy, we quantified Sls and Projectin ends by combining oligo-nucleotide–labeled nanobodies with DNA-PAINT, a technique that achieves about 5-nm spatial resolution in flight muscle tissue (*25*). This not only verified the increased sarcomere length in *sls[23-extraPEVK-YPet]* but also showed that Projectin N and C-termini are both only about 20 nm further away from the Z-discs compared to controls (Fig. 3, G and H), and hence, the total I-band length increases only by twice 20 nm. This is also supported by directly measuring the position of the C-terminal end of Sls (Fig. 3, G and H). Together, these data show that adding the same extra spring to the I-band titin Sls has muscle type–specific effects: It elongates the larval muscle sarcomeres by 1 μm but the flight muscle sarcomeres by less than 200 nm. In both cases, the A-band rescales to the changes in I-band length.

Does this change in sarcomere length have functional consequences? *Drosophila* flight muscles oscillate faster than 200 Hz during flight, while sarcomeres shorten less than 2% (*29*). This requires a very stiff muscle to respond mechanically very quickly. Adding a PEVK spring to Sls in flight muscles should make the sarcomeres more compliant; as a consequence, the longer I-band should lower the oscillation frequency of the wings because the resonance frequency of the system is determined by the stretch activation of the contractile muscle elements and the inertia of the moving load, as demonstrated by the increase in the oscillation frequency by reducing the size of the wings (*30*, *31*). To quantify wing beat frequency, we recorded the sound generated from *sls[23-YPet]* and *sls[23-extraPEVK-YPet]* flies during tethered flight (fig. S5A and see the Materials and Methods). This revealed a lower wing beat frequency in *sls[23-extraPEVK-YPet]* males compared to *sls[23-YPet]* control (Fig. 3I and fig. S5B). To test the consequences of lower wing beat frequency, we performed flight assays and found that flight capability is impaired in *sls[23-extraPEVK-YPet]* males and females (Fig. 3J). This provides a functional explanation for why *Drosophila* flight muscles do contain a short and stiff version of the I-band titin Sls.

### Titin forces correlate with muscle type–specific sarcomere length

Why does inserting the same extra PEVK part of Sls result in sarcomere type–specific effects on Sls length? This hints at important muscle type–specific differences in the forces across Sls. To obtain a first estimate of molecular forces across muscles, we used established force sensors in the integrin adaptor protein Talin in larval muscles, allowing us to measure forces at the muscle-tendon junction (*32*). Comparing control and intramolecular force sensors, we found that about 30% of the Talin molecules experience forces above 10 pN in relaxed larval muscles (fig. S6, A and B). These forces are not changed in *sls[*∆*31-32]*. However, they are larger compared to forces across Talin at adult flight muscle attachment sites (*32*), which suggests that passive stretching forces in relaxed larval sarcomeres are indeed higher compared to flight muscles.

To directly quantify the stretching forces across I-band titin, we inserted these Förster Resonance Energy Transfer (FRET)-calibrated molecular force sensors into exon 23 and exon 32 of Sls, generating *sls[23-TS]*, *sls[23-stTS]*, *sls[32-TS]*, and *sls[32-stTS]*, respectively, as well as the controls *sls[23-mCherry]*, *sls[23-YPet]*, *sls[32-mCherry]*, and *sls[32-YPet]* (figs. S1B and S4C, and see Material and Methods). All *sls* sensor flies are homozygous viable, demonstrating the functionality of the internally tagged Sls proteins. However, despite multiple efforts, we failed to generate a Sls C-terminal no-force control sensor knock-in line.

**Fig. 4.**
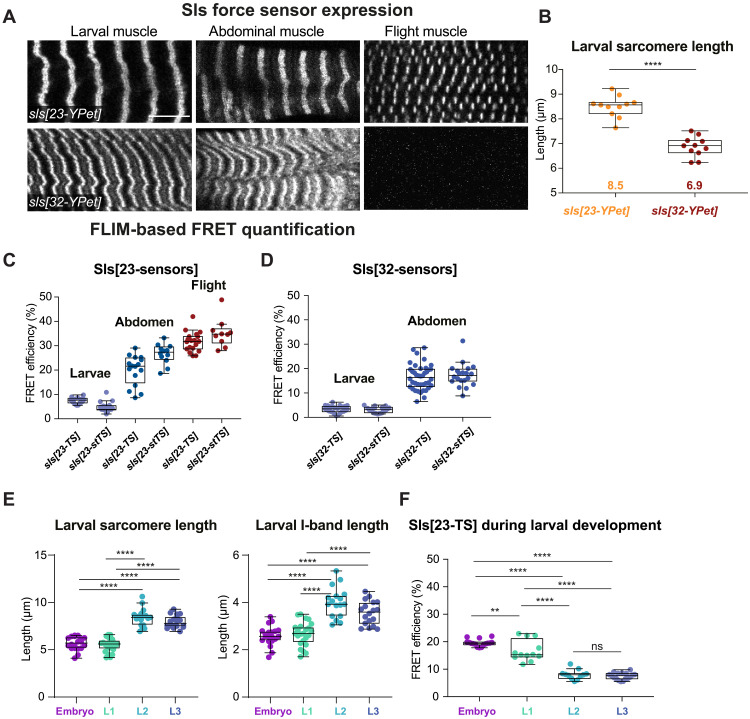
Sls molecular forces in different muscle types in vivo. (**A**) In vivo expression of *sls[23-YPet]* and *sls[32-YPet]* in third instar larval, adult abdominal, and adult flight muscles. Scale bar, 10 μm. Note that *sls[32-YPet]* is not expressed in flight muscles. (**B**) Quantification of sarcomere length in heat fixed third instar larval VL3 muscles of *sls[23-YPet]* (*N* = 22) and *sls[32-YPet]* (*N* = 22)*.* Mann-Whitney test, ****P < 0.0001. Plots are Tukey’s box and whisker plots with median as lines, and first quartile and second quartile as box and whisker size are 1.5 times the IQR. All animals measured are represented as dots. (**C** and **D**) FLIM-based FRET quantification of *sls[23-TS]* and *sls[23-stTS]* (C) and *sls[32-TS]* and *sls[32-stTS]* (D) in larval, abdominal, or flight muscles (*n* ranges from 10 to 40 per muscle and genotype, see source data S4). (**E**) Sarcomere length and I-band length (calculated by measuring the A-band length; see fig. S6E) in alive MhcGFP expressing embryos (*N* = 20) and larvae at different larval instars (L1, *N* = 24; L2, *N* = 17; L3, *N* = 18). Tukey’s multiple comparisons test, ****P < 0.0001. (**F**) FLIM-based FRET quantification of *sls[23-TS]* in alive embryos (*N* = 16) and different larval instars (L1, *N* = 12; L2, *N* = 11; L3, *N* = 17). Tukey’s multiple comparisons test, **P < 0.01 and ****P < 0.0001.

As expected from the mRNA expression data (fig. S1A), all sensors and controls inserted in *sls* exon 23 are expressed in all muscle types, while the ones in exon 32 are not expressed in flight muscles (Fig. 4A). Because we replaced *sls* exon 32 (containing a large PEVK region) with the sensor, the larval sarcomere length of *sls[32-YPet]* is reduced by 1.5 μm, similar to what we had found in *sls[32-V5]* and *sls[*∆*31-32]* (Fig. 4B).

To better characterize these Sls FRET tension sensors, we first measured intermolecular FRET by quantifying the fluorescence lifetime of the YPet donor in the presence or absence of the respective Cherry acceptor in different Sls molecules in trans. We found that intermolecular FRET is negligible (fig. S6, C and D). Hence, we measured intramolecular FRET using *sls[23-TS]* and *sls[23-stTS]* in the different muscle types and found very high FRET values in flight muscles comparable to the no-force control values in the Talin sensor (*32*), suggesting that stretching forces across Sls (the molecular tension) in resting flight muscles are lower than 8 pN, as the sensor does not open, thus FRET is high (Fig. 4C). The same sensors display very low FRET in larval muscles and intermediate FRET in abdominal muscles suggesting large stretching forces (>12 pN) across Sls in relaxed larval and intermediate forces in the abdominal muscles (Fig. 4C). A similar difference is found in *sls[32-TS]* and *sls[32-stTS]* in larval versus abdominal muscles (Fig. 4D). This is consistent with the hypothesis that long I-band sarcomeres experience high passive forces, stretching Sls to more than 2 μm in length. High Sls stretching forces are also supported by the above finding that the deletion of 1700 amino acids, coded in the PEVK-rich exon 32 of *sls*, reduces Sls protein length by about 0.5 μm, consistent with a fully unfolded conformation of the PEVK-rich sequence [contour length per amino acid: about 0.35 nm (*33*); 1700 × 0.35 nm = 595 nm].

To investigate if these large forces are already present when the larval muscles assemble sarcomeres at the end of embryogenesis (*23*), we measured FRET across *sls[23-TS]* in stage 17 embryos, as well as in first and second instar larvae. We indeed find higher FRET values in embryos and L1 larvae, indicating lower forces, which interestingly correlate with their shorter sarcomere, I-band, and A-band length (Fig. 4, E and F, and fig. S6E). These findings support a force-feedback mechanism, in which forces across Sls scale the I-band, which in turn scales the A-band, and thus control the dimensions of the sarcomere.

### A biomechanical feedback model of sarcomere filament length control

**Fig. 5.**
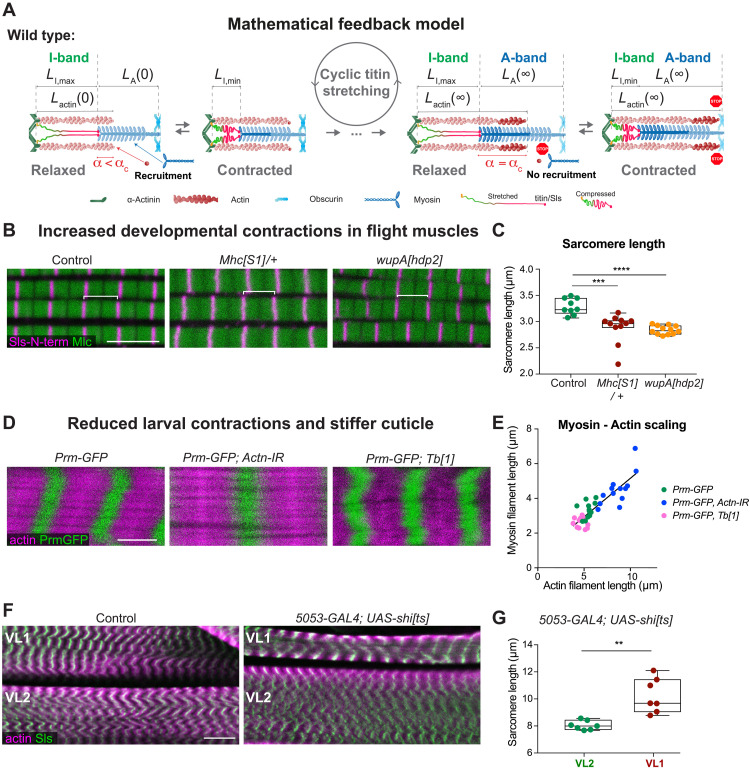
A titin-instructed biomechanical feedback controls filament length. (**A**) Mathematical model of a Sls instructed biomechanical feedback regulating I-band and A-band length: Actin and myosin filaments grow until a constant actin/myosin overlap (α_C_) is reached in the relaxed state (stretched titin). If in the relaxed state α < α_C_, actin and myosin filaments can grow at their ends (highlighted by darker colors). If α = α_C_ is reached in the relaxed state, then growth stops as frequent contractions are initiated and then recruitment in the contracted state is blocked (red STOP signal). Such a stable A-band length is reached that scales with the I-band length. See section “A biomechanical feedback model” and the Materials and Methods for details. (**B** and **C**) Wild-type control (*N* = 9) and hypercontractile *Mhc[S1]/+* (*N* = 12) and troponin I (*wupA[hdp2]*, *N* = 11) mutant flight muscle sarcomeres were stained with Sls-N-terminal to label the Z-discs (magenta) and anti-myosin light chain nanobody (NB1-Mlc1) to label myosin (green). Scale bar, 5 μm. (C) Sarcomere length from (B). Tukey’s multiple comparisons test, ****P < 0.0001. (**D** and **E**) Larval sarcomeres of wild type (*Prm-GFP, Mef2-GAL4*; *N* = 12), *Actinin* knockdown (*Prm-GFP*, *Mef2-GAL4*, *Actn-IR*; *N* = 12), or *Prm-GFP Tubby* (*Tb[1]*; *N* = 16) stained for actin (phalloidin in magenta) and myosin length (Prm-GFP, green). Scale bar, 5 μm. (E) Actin and myosin length quantification shows the scaling of both. (**F** and **G**) Third instar larval sarcomeres of wild type and *5053-GAL4, UAS-shi[ts]* shifted to the restricted temperature at L1 stage. Scale bar, 20 μm (G). Quantification of (F) (*N* = 8). Mann-Whitney test, **P < 0.01.

To gain a deeper mechanistic understanding of the molecular mechanism of the feedback, we propose a simple mathematical model that quantitatively explains the scaling of the myosin filament length to that of the extended titin molecules and thus to forces present in the muscle type (Fig. 5A). We consider that the sarcomere undergoes cycles of contraction (of duration *T*_c_) and phases of relaxation (of duration *T*_r_). We express the problem in terms of three characteristic lengths—*L*_A-band_(*t*), *L*_I-band_(*t*), and L_actin_(*t*)—which correspond to the length of the myosin filaments, titin molecules, and actin filaments, respectively. During a contraction phase, the myosin motors compress titin up to an equilibrium length, denoted *L*_I,min_. Within titin’s linear elasticity range, such length is *L*_I,min_ = *L*_I,max_ − *T*_motor_/*K*_titin_, with *T*_motor_ being the maximal compression force and *K*_titin_ is the titin elastic modulus. During a relaxation phase, the I-band relaxes to its long equilibrium length, *L*_I-band_(*t*) = *L*_I,max_. Our model proposes that during phases of muscle relaxation, new actin and myosin subunits can be added to the filament ends, resulting in a growth of the filaments. In sarcomeres with long I-bands (high force or long titin), the accessibility of myosin motors to the actin filaments is limited, lowering the strength of contraction. This promotes the recruitment of new actin and myosin subunits, which increases filament length; in turn, once the actin filaments have grown up to a length that exceeds sufficiently that of the I-band, an increasing number of myosin motors can bind; once this number *N*_motors_ exceeds a critical value *N*_c_, the sarcomeres contract at high force (*34*), which blocks new subunit recruitment. This translates into a maximum actin filament length *L*_actin_ = *L*_I,max_+ α, with α = *N*_c_ l and l as the myosin filament length, beyond which the sarcomere contracts. In turn, this maximum actin filament length caps the maximal length of the myosin filaments set during the contraction phase at *L*_A-band_(∞) = *L*_I,max_ + α − *L*_I,min_. Using this model, we find that the final length of the A-band [*L*_A-band_(∞) = *L*_I,max_ + α − *L*_I,min_] and the actin filaments [*L*_actin_(∞) = *L*_I,max_ + α] scale with the relaxed length of the I-band (*L*_I,max_) (Fig. 5A). Thus, smaller I-band titin molecules or lower titin forces result in a shorter I-band and in turn instruct a shorter A-band (fig. S7, A and B). This is consistent with our above in vivo findings.

This model predicts that reducing the sarcomere contraction strength will increase sarcomere length, as well as the actin and myosin filament length, because the time to grow the filaments at the relaxed stage is increased (fig. S7, A and C). This scenario is at play during flight muscle development and results in the observed long actin and myosin filaments with 95% overlap despite having a short I-band: at 48 hours after puparium formation (APF) sarcomeres are 2 μm short and spontaneously contract. After 48-hour APF, these contractions stop until 90-hour APF when flies eclose with 3.4-μm-long flight muscle sarcomeres that have 3.2-μm-long myosin filaments (see Fig. 3) (*24*). In various mutants, in which the spontaneous muscle contractions continue after 48-hour APF, the sarcomeres remain shorter at 90-hour APF (*24*, *35*–*38*). Strikingly, we found that sarcomeres of particular Myosin heavy chain (*Mhc*) or Troponin I (*wupA*) mutations, which cause excessive contractions during development (*39*, *40*), are abnormally short, containing shorter myosin filaments (Fig. 5, B and C). Thus, our force-feedback mechanism can explain how sarcomeres with a maximum actomyosin overlap (a longer α_c_ in the model fig. S7, A and C) are made during development. These powerful sarcomeres can oscillate at high frequency to mediate insect flight.

To explore if filament growth can also be induced ectopically in sarcomeres that do readily contract, we choose the larval muscles and knocked-down α*-actinin*, which strongly reduces larval locomotion and thus sarcomere contractility (fig. S7, D and E, and movie S1). Consistent with our proposed feedback, we found that not only sarcomere length but also actin and myosin filament length are increased in these slowly moving larvae (Fig. 5, D and E, and fig. S7, D to G).

To further challenge the model, we blocked sarcomere contractions in a single muscle cell per larval hemisegment only by expressing dominant negative Dynamin (*shi[ts]*) (see the Materials and Methods) (*41*). Strikingly, the silenced muscle VL1 displays elongated sarcomeres with longer actin filaments compared to its direct VL2 neighbor analyzed in the same larva (Fig. 5, F and G, and fig. S7H). This demonstrates that extended periods of muscle relaxation result in an elongation of actin and myosin filaments, as predicted by our model, and hence result in longer sarcomeres without the need for a gigantic titin A-band ruler protein.

Last, we aimed to mechanically manipulate the muscle independently of the sarcomere components. To do so, we took advantage of a dominant mutation in the *Tubby* gene (*Tb[1]*) that results in short and fat larvae. As Tb is a chitin-modifying enzyme, likely the exoskeleton is more rigid, resulting in the short pupae (*42*, *43*). We confirmed that sarcomeres in these Tubby larvae are shorter, as had already been shown (*44*), and we found that both actin and myosin filaments are shorter, too. Strikingly, both filaments scale linearly in all the genotypes in larval muscles (Fig. 5, D and E, and fig. S7G). This demonstrates that changes in the exoskeleton feedback on the sarcomere components to adjust filament length.

**Fig. 6.**
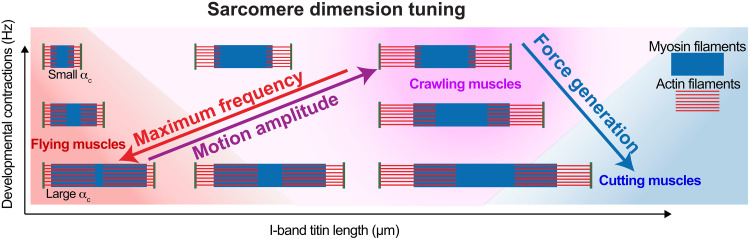
A sarcomere type phase diagram. A phase diagram that illustrates how two variables, developmental muscle contractions (resulting in changes of actin-myosin overlap α_C_) and I-band titin length, can generate sarcomeres with vastly different dimensions, which are specialized for maximum frequency at high force (flying muscles, red), large motion amplitude (crawling muscles, magenta), or maximum force generation (cutting muscles, blue).

Together, we have uncovered a biomechanical mechanism to regulate the dimension of the sarcomere in the different *Drosophila* muscle types that is both genetically and mechanically controlled. The length of the I-band titin and developmental contractions can tune the filament and sarcomere length to allow the formation of very stiff muscles with an extensive actomyosin overlap, which oscillate at high frequency to power insect flight. In the same organism, much more compliant muscles, which contract slower but at higher amplitude, to support larval crawling and adult walking can be constructed (Fig. 6). Both would not be compatible with the strict titin ruler that is applied in mammalian sarcomeres.

### Evolutionary conservation

Is the force-feedback mechanism conserved outside of insects? Our titin evolutionary tree revealed two distinct titin homologs not only in beetles but also in nematodes (Fig. 1, A and B), suggesting that a similar mechanism may control actin and myosin filament length in the sarcomeres of a wide variety of non-vertebrate species.

To investigate the sarcomere structure of the nematode *C. elegans*, we used electron microscopy. This revealed the unique tilted arrangement of the actin and myosin filaments in *C. elegans* body muscle sarcomeres, which makes length quantifications difficult (*45*). Sectioning adult *C. elegans* in the plane of the myosin filaments revealed a sarcomere length of about 18 μm containing very long (about 15 μm) myosin filaments (Fig. 1,D, D′). As the A-band titin homolog Twitchin is only about 7000 amino acids long and, similar to Projectin in *Drosophila* larval muscles (*23*), decorates the entire myosin filament (*46*), a classical titin ruler mechanism of myosin filament length control is neither possible in nematodes nor in insects. Thus, we have uncovered how the vastly different sarcomere dimensions present in a large clade of the animal tree are set in a controlled way to match the biomechanical requirements of specialized tailor-made muscle types.

## DISCUSSION

The here revealed force-feedback mechanism controlling the length and architecture of the key sarcomere filaments provides a molecular explanation for how animals can produce tailor-made sarcomeres for specialized muscle functions. By applying this mechanism, sarcomeres tuned for fast oscillations powering insect flight and sarcomeres tailored for large motion amplitudes such as walking or larval crawling or highest force production to cut fibrous leaves can be built within the same animal.

In addition, the feedback mechanism equips the muscles with more flexibility. It allows an animal to adapt its muscles to changes in their mechanical environment, for example, during larval molding or to variations in the mechanical properties of the surrounding medium. It can also adjust to physiological changes during ageing or to changes of the animal’s internal state. One important physiological adaptation occurs in females after mating to effectively produce eggs. Mated females eat more and their gut enlarges in diameter (*47*–*49*). As a consequence, their circular muscles need to grow in length to cover the larger gut surface. Smartly, *Drosophila* females solve this problem by increasing their actin, myosin, and consequently sarcomere length after mating (*50*), likely using the here proposed mechanism as guts in mated females contract less frequently (*50*). This allows females to absorb food more effectively and thus produce more eggs. The feedback also readily enables evolutionary adaptions to variable thorax sizes and wing beat frequencies, for example, the slow wing beat frequencies found in large butterflies. In summary, the here described force-feedback mechanism allows muscles to specialize and adapt to the particular tasks, which may be one of the key reasons for the success of insects and nematodes during evolution.

As a consequence of the vastly different sarcomere dimensions, including variations of the myosin filament length, a strict titin ruler mechanism, as present in mammals, cannot be applied. This would need a gigantic A-band titin gene, much larger than the mammalian one. The mechanism that tunes actin and myosin filament growth and subunit incorporation rates are likely adjusted by additional muscle type–specific signals than only contraction rates. For example, insect flight muscles contain particular isoforms of the myosin filament binding proteins Myofilin, Paramyosin, and Stretchin that are different from the larval or leg muscle isoforms (*26*, *51*, *52*). Furthermore, actin filament capping or actin polymerization promoting protein expressions vary between muscle types, too (*24*, *53*). It is enticing to speculate that tuning these various parameters will instruct the feedback mechanism to build bespoke muscle types with biomechanical properties for optimized mechanical output (*54*).

## MATERIALS AND METHODS

### Experimental design

#### Drosophila *strains and genetics*

Fly stocks were maintained under standard culture conditions at 27°C (*55*). All new *sls* alleles generated in this study are listed in fig. S1B. The *Zasp66-GFP* allele was used to label the Z-disc (*56*); *Prm-GFP* (fTRG-475) to label the A-band (*52*); *Mhc[weepGFP]* to label a subset of Mhc isoforms (*57*); and *Talin-YPet*, *Talin-TS*, and *Talin-C-TS* to quantify force across Talin (*32*). The CRISPR injections were done into *Act5c-Cas9, DNAlig4[169]* (*32*). Heat shock flippase was used to generate *sls[*∆*31-32]*), and *Df(3 L)BSC366* was used as *sls* deficiency (both from Bloomington). The *Actn* RNAi line (V7760) was from Vienna Drosophila Resource Center (VDRC) (*58*). The *Mhc[S1]* allele was from Troy Littleton (*39*) and the *wupA[hdp2]* (Troponin I) allele from A. Cammarato (*40*). Canton S or *w[1118]* were WT controls.

#### Titin evolutionary tree

Human titin (NP_001243779.1) and *Drosophila melanogaster* Sallimus (NP_001261304.1) and Projectin/bent (NP_001162825.1) were downloaded from the National Center for Biotechnology Information Protein database and used with BLAST (*59*, *60*) to search for reciprocal best hits (RBHs) (*61*) with Deuterostoma and Protostoma and their subphyla, respectively. Because of its length and its repetitive nature, titin may be mis-sequenced or confused with obscurins. Thus, we manually selected only orthologs we found to have strong evidence based on the quality of available sequencing data. Thus, we kept the 15 species presented in Fig. 1A. Their protein identifiers are listed in table S1 and the sequences are in file S1.

We used InterPro (*62*, *63*) to predict the different domains in each ortholog. Using a Python3 script, we curated the results, keeping only our domains of interest, i.e., Ig, Fn3, and kinase domains and Z-repeats. Although InterPro can calculate the presence of PPAK motifs, repeated motifs specific to PEVK domains, we calculated the presence of these PEVK domains with a Python3 script, which calculates the proportion of P, E, V, and K residues in a given window of 250 amino acids. Windows with more than 40% of P, E, V, and K were considered as PEVK domains. The Python script is also attached in file S1. In the end, the proteins were represented using R with respect to their sizes, domains, and PEVK regions. As the orthologs are very different, it was impossible to build a phylogenetic tree. We thus decided to use the common species tree proposed by the NCBI taxonomy (*64*) for visualization purposes.

#### CRISPR and RMCE

Modification of Sls length and the insertion of tension sensors were made by combining CRISPR-Cas9–mediated genome engineering with phiC31-mediated cassette exchange as described (*65*). Briefly, the genomic sequence in the *Act5c-Cas9, DNAlig4[169]* was sequenced, the single guide RNAs (sgRNAs) were designed with an online tool (https://crisprscan.org) and transcribed in vitro (MEGAshortscript T7 Kit, Invitrogen). The efficiency of the sgRNAs was tested by embryo injection into *Act5c-Cas9* or in Cas9-expressing S2 cells with a T7 endonuclease assay (*65*). For step 1, one pair of sgRNAs (100 ng/μl) was injected into *Act5C-Cas9, DNAlig4[169]* embryos in combination with a dsRed donor vector (500 ng/μl) containing a dsRed eye marker cassette flanked by attP sites and homology arms (see figs. S2 and S4). Successful homologous recombination events were identified by red fluorescent eye color and verified by sequencing. For step 2, vasa-phiC31 plasmid (200 ng/μl) was injected into step 1 embryos together with an attB-donor vector (150 ng/μl). Successful exchange events were identified by the absence of red fluorescent eyes, and the correct orientation of the cassette was verified by polymerase chain reaction. Homozygous stocks were established from single flies.

#### Flight and leg muscle staining

Detailed flight and leg muscle staining protocols were recently published (*23*, *66*). Briefly, the head, abdomen, and wings were clipped from young adult flies (3 to 7 days old), and thoraces were fixed in 4% paraformaldehyde (PFA) in PBS-T (phosphate-buffered saline with 0.3% Triton X-100) for 20 min at room temperature. After washing once with PBST, thoraxes were placed on a slide with double sticky tape with the anterior facing the tape and cut sagittally with a microtome blade (Pfm Medical Feather C35). Hemi-thoraces were stained with fluorescent nanobodies and rhodamine-phalloidin (1:1000; Molecular Probes) for 2 hours at room temperature (RT) or overnight at 4°C. Hemi-thoraces were washed twice with PBS-T, mounted in SlowFadeTM Gold Antifade (Thermo Fisher Scientific) using two coverslips as spacers, and flight or leg muscles were imaged with a Zeiss LSM880 confocal microscope using a 63× objective.

#### Dissection and staining of larval muscles

A detailed protocol for staining of larval muscles was published (*23*). Briefly, third instar (L3) larvae were either immobilized by dipping for 1 s in 65°C water (*67*) or alive covered with HL3 buffer (70 mM NaCl, 5 mM KCl, 1.5 mM CaCl_2_, 20 mM MgCl_2_, 10 mM NaHCO_3_, 5 mM trehalose, 115 mM sucrose, and 5 mM Hepes) (*23*), pinned individually, and dissected with sharp scissors from the dorsal side. Interior organs were removed with forceps, and the larval filets were fixed in 4% PFA in PBS-T (PBS with 0.3% Triton X-100) for 30 min and then blocked in 4% normal goat serum for 30 min at room temperature. Nanobodies and antibodies were incubated in PBS-T overnight at 4°C. Larval filets were then washed three times 10 min in PBST at RT and stained with secondary antibodies and phalloidin (1:1000; labeled with rhodamine, Molecular Probes) in PBS-T for 2 hours at RT in the dark. After washing three times with PBST for 5 min, larval filets were mounted in SlowFadeTM Gold Antifade (Thermo Fisher Scientific) and imaged with a Zeiss LSM880 confocal microscope using 20×, 40×, or 63× objectives.

#### Antibodies and nanobodies

Primary antibodies [anti-Mhc (3e8-3D3, mouse, DSHB; 1:100), anti-V5 (mouse, Invitrogen; 1:1000)] or fluorescently labeled nanobodies [Sls-Nano2 (1:1000), Sls-Nano39 (1:1000), Sls-Nano42 (1:1000), Sls-Nano48 (1:1000), and Proj-Nano37 (1:1000) (*23*)] were incubated overnight at 4°C. After washing three times 10 min in PBST at RT, samples were stained with secondary antibody (1:500; Alexa488 goat anti-mouse IgG, Molecular Probes) and/or phalloidin (1:500; rhodamine conjugate, Molecular Probes) in PBST for 2 hours at RT in the dark. The newly made anti-myosin light chain nanobody NB1-Mlc1 was generated by immunizing a trimeric complex of Mlc1, Mlc2, and a cognate myosin heavy chain fragment (comprising residues 709 to 840). This complex was generated by coexpressing the three proteins as His14-ScSUMO and His14-NEDD8 fusions in *Escherichia coli* and purifying them by capture to Ni-chelate beads followed by a tag-cleaving elution with Ulp1 and NEDP1 (*68*). Immune library generation, phage display, nanobody production, and nanobody labeling were performed as described (*23*, *69*). Biolayer interferometry measurements revealed that the nanobody targets the Mlc1 subunit with a *K*_D_ of ~1 nM.

#### Image quantification and filament length measurements

Quantifications of protein or filament lengths and distance between proteins were achieved using the automated algorithms of the Fiji macro toolset PatternJ (*28*, *70*). In brief, after the manual selection of a myofibril in Fiji, the resulting myofibril intensity profile is used by the PatternJ algorithms to extract the position of bands or edges of filaments in the following way:

1. The user specifies to PatternJ the main features of protein stainings in a sarcomere from predefined patterns (one or multiple bands, blocks, and blocks with bands).

2. PatternJ obtains the average sarcomere length using the autocorrelation of its intensity profile. Based on the position of the highest intensity in the profile, it defines a reference sarcomere.

3. It extracts the position of all sarcomeres using a cross-correlation between the intensity profile of the myofibril and the intensity profile of the reference sarcomere, allowing for the segmentation of each sarcomere.

4. Once each sarcomere is segmented, particular features defined in the first step are then located and localized precisely by fitting with relevant mathematical functions, such as a Gaussian function for a thin band or a spline function to locate the edge of a filament.

Based on the position of the features extracted with PatternJ, we straightforwardly obtained the different lengths of filaments or distances between bands.

#### DNA-PAINT

For DNA-PAINT, adult flies were fixed as described above for immuno-stainings and hemi-thoraces were stained and imaged as described recently (*25*). Briefly, Sls-Nano39, Proj-Nano29, or Proj-Nano37 coupled to P3 oligos (*25*) (about 50 nM) were incubated with fly hemi-thoraces overnight. After washing with PBS and 1% Triton, stained hemi-thoraces were mounted in the imaging chamber, and the respective imager oligos coupled with Cy3B (Metabion) were added in imaging buffer, buffer C (1× PBS and 500 mM NaCl), at 2 nM. Directly before imaging, buffer C was supplemented with 1× Trolox, 1× 3,4-dihydroxybenzoic acid (protocatechuic acid, PCA), and 1× protocatechuate 3,4-dioxygenase pseudomonas (PCD). 100× Trolox: 100 mg of Trolox, 430 μl of 100% methanol, 345 μl of 1 M NaOH in 3.2 ml of H_2_O. 40× PCA: 154 mg of PCA, 10 ml of water, and NaOH were mixed, and pH was adjusted to 9.0. 100× PCD: 9.3 mg of PCD, 13.3 ml of buffer [100 mM tris-HCl (pH 8), 50 mM KCl, 1 mM EDTA, and 50% glycerol]. All three were frozen and stored at −20°C. Total internal reflection fluorescence (TIRF) imaging of the single-molecule DNA hybridization events was done on a TIRF illumination system (SAFE 360, Abbelight, France) combined with an inverted microscope using a 100X numerical aperture 1.47 objective (Evident, Japan). Coverslips with embedded gold particles were used for drift correction (550-100Auf, Hestzig LLC). Images were processed, and bands were fitted as described in detail recently (*25*).

#### Behavioral tests

Tethering and flight frequency recording: male flies were anesthetized on ice, then moved to a Peltier-cooled aluminum block kept at 4°C, and fixed with their notum onto a tungsten pin with ultraviolet hardening glue (Tetric EvoFlow, Ivoclar Vivadent), as described previously (*71*, *72*). The wire was then fixed on a block of plasticine to suspend the male (fig. S5A). The tethered fly was provided with a polystyrene ball (diameter of 2 to 3 mm) as support and then placed above an electret condenser microphone (CMP-5247TF-K, CUI Inc) (distance of 2 cm). After a recovery of approx. 1 min, the ball was removed with a gentle air puff, triggering flight. The flight was recorded for 10 min, amplified with a custom-made circuit board, and digitized with a multifunction data acquisition device (NI USB-6259 MASS Term, National Instruments) (*72*, *73*).

Flight wing beat frequency analysis: analysis of the flight wing beat frequency from recorded signals was performed with Raven 1.6 (The Cornell Lab of Ornithology) using a fast fourier transform–type Hann, with a window length of 10,000 samples and 0% overlap. The sound was filtered with a bandpass filter (between 150 and 350 Hz) to exclude harmonics beside the fundamental band. For each flight bout, the first seconds were excluded from the analysis to avoid possible interference due to the air puff used to trigger flight.

To quantify flight behavior, about 30 male flies (1 to 3 days old, aged at 25°C) were sorted into a fresh food vial and aged for another 24 hours. Then, they were thrown through a funnel at the top of a 1 m × 8 cm plexiglass cylinder with five marked sections according to an established protocol (*67*). The landing positions of the flies were noted. Flight assays were performed in triplicates with a total of at least 80 to100 males tested.

To quantify larval locomotion, we used a recently published protocol (*23*). Briefly, third instar (L3) larvae were collected at the wandering stage, placed on an apple agar plate, and allowed to acclimatize for at least 20 min at room temperature. Larvae were then placed simultaneously in the center of the plate and imaged at a frame rate of 25 Hz. Images were acquired using an infrared Basler acA2040-90μmNIR camera equipped with a Kowa LM12SC lens and a homemade light-emitting diode (LED) infrared illumination system (WINGER WEPIR3-S1 IR Power LED Star infrared at 850 nm). The Pylon viewer software from Basler was used to control acquisition, and exposure time was adjusted for enhanced contrast. The assays were repeated at least three times for each genotype, with assays done on different days. The videos were analyzed using FIMTrack (*74*), and data were visualized via Python.

#### FLIM-FRET quantification

The FLIM data were acquired on Zeiss LSM880 confocal equipped with the PicoQuant LSM Upgrade Kit using a PicoQuant Time-Correlated-Single-Photon detector [TimeHarp 260, in version 250 ps (NANO module) base resolution] and a femtosecond pulsed 510-nm laser running at a repetition rate of 100 MHz. The data were analyzed using the SymPhoTime 64 software (PicoQuant). First, an intensity image was created by manually drawing an region of interests (ROI) around the target structure (Z-discs or muscle attachment sites). Second, photon arrival times of all photons inside the ROI were plotted in a histogram, and the tail of the curve was fitted with a mono-exponential decay fit for the YPet donor alone or with a bi-exponential decay fit for all the different tension sensors to calculate an average fluorescence lifetime τ. Third, the FRET efficiency (*E*) for each tension sensor was calculated according to the following formula, with τ_DA_ being the lifetime of the donor in the presence of the acceptor (the tension sensor pair) and τ_D_ the lifetime of the donor alone (YPet control)

$$E=1-\frac{\tau_{\text{DA}}}{\tau_{D}}$$

For all measurements, τ_D_ was determined as the median lifetime of either Talin [YPet], Sls[23-YPet], or Sls[32-YPet] using the same experimental conditions. Experiments were repeated more than three times on different experiment days.

#### *Electron microscopy of* C. elegans *muscles*

N2 worms were frozen in 50 mM NaCl medium containing 5% of bovine serum albumin and *E. coli* bacteria using Leica EM Pact 2 high-pressure freezer. After freezing, samples were freeze-substituted at −90°C in acetone containing 2% OsO_4_ for 96 hours. The temperature was gradually increased to −60°C and maintained for 8 hours. The temperature was then raised to −30°C and maintained for 8 hours, before to be raised to RT. Samples were finally washed in acetone and embedded in epoxy resin. Resin was polymerized at 60°C for 48 hours. Then, ultrathin sections (70 nm) were made using a Leica UC7 ultramicrotome and post-stained with 2% uranyl acetate and Reynolds’ lead citrate. Images were taken with a Tecnai G2 microscope [Field Electron and Ion Company (FEI)] at 200 kV using the Photomontage module to obtain the tiles. The montage was produced with the Blendmont script from the eTomo suite.

#### Mathematical modeling

In our simulations, we consider that the sarcomere unit undergoes cycles of contraction (of duration *T*_c_) and phases of relaxation (of duration *T*_r_). The system evolves linearly within each phase, with

$$L_{\text{actin}}\left(t\right)=\text{max}[L_{\text{actin}}\left(T_{n,r}\right)+v_{\text{actin},r}\;\left(t-T_{n,r}\right),L_{0}+\alpha ]$$

$$L_{A‐\text{band}}\left(t\right)=L_{A‐\text{band}}\;\left(T_{n,r}\right)+v_{A,r}\;\left(t-T_{n,r}\right)$$

$$L_{I‐\text{band}}\left(t\right)=L_{I,\text{max}}$$

during the n-th relaxation phase, with *T*_n,r_ as the start date of the last relaxation phase and *v*_r_ as the actin and A-band growth speed in the relaxation phase, respectively, and

$$L_{\text{actin}}\left(t\right)=\text{min}\left[L_{\text{actin}}\left(T_{n,c}\right)+v_{\text{actin},c}\;\left(t-T_{n,c}\right),L_{c}+L_{A‐\text{band}}\;\left(T_{n,c}\right)\right]$$

$$L_{A‐\text{band}}\left(t\right)=L_{A‐\text{band}}\;\left(T_{n,c}\right)$$

$$L_{I‐\text{band}}\left(t\right)=L_{I,\text{min}}$$

during the n-th contraction phase, with *T*_n,c_ as the date of the start of the last contraction phase. Because of the wall condition at the center of the sarcomere, the actin filaments cannot exceed the sum of the titin and myosin lengths *L*_actin_(*t*) < *L*_I-band_(*t*) + *L*_A-band_(*t*) (see Fig. 5A); we make sure that our initial conditions verify this condition. We then solve the system of equations numerically and consider the final state reached. As expected (see main text), both the final lengths of the actin [*L*_actin_(∞) = *L*_I,max_ + α] and A-band [L_A-band_(∞) = *L*_actin_(∞) − *L*_I,min_ = *L*_I,max_+ α − *L*_I,min_] scale with the relaxed length of the I-band (*L*_I,max_). The used code is available in data S2.

### Statistical analysis

Graphs and statistical analysis were made with Prism (GraphPad). Mann-Whitney tests (between two groups), Tukey’s multiple comparison tests (between multiple groups), or Chi-square tests (between multiple bin distributions) were used. The plots shown in the figures are Tukey’s box and whisker plots with median as lines, first quartile and second quartile as box, and whisker size is 1.5 times the interquartile range. All animals measured are represented as dots, and average values are noted as numbers in some plots to make it easy for the reader to appreciate the average value calculated. The values for *N*, *P*, and the specific statistical test performed for each experiment are included in the appropriate figure and figure legend as well as in the source data table containing all the measurements used for the shown analysis.

## Supplementary Materials

### The PDF file includes:

Figs. S1 to S7 Legend for movie S1 Legend for table S1 Legends for data S1 to S5 Legends for files S1 and S2
