# Supplementary material for: Titin-dependent biomechanical feedback tailors sarcomeres to specialized muscle functions in insects
Source: Sci Adv. 2025 May 9;11(19):eads8716. doi: 10.1126/sciadv.ads8716 (PMC12063666; doi:10.1126/sciadv.ads8716)
Supplement: Supplementary file 1 — Figs. S1 to S7 Legend for movie S1 Legend for table S1 Legends for data S1 to S5 Legends for files S1 and S2 [file sciadv.ads8716_sm.pdf]

Science Advances
AAAS

Supplementary Materials for

Titin-dependent biomechanical feedback tailors sarcomeres to specialized muscle functions in insects

Vincent Loreau *et al.*

Corresponding author: Frank Schnorrer, frank.schnorrer@univ-amu.fr

Sci. Adv. **11**, eads8716 (2025)
DOI: 10.1126/sciadv.ads8716

The PDF file includes:

Figs. S1 to S7
Legend for movie S1
Legend for table S1
Legends for data S1 to S5
Legends for files S1 and S2

Other Supplementary Material for this manuscript includes the following:

Movie S1
Table S1
Data S1 to S5
Files S1 and S2

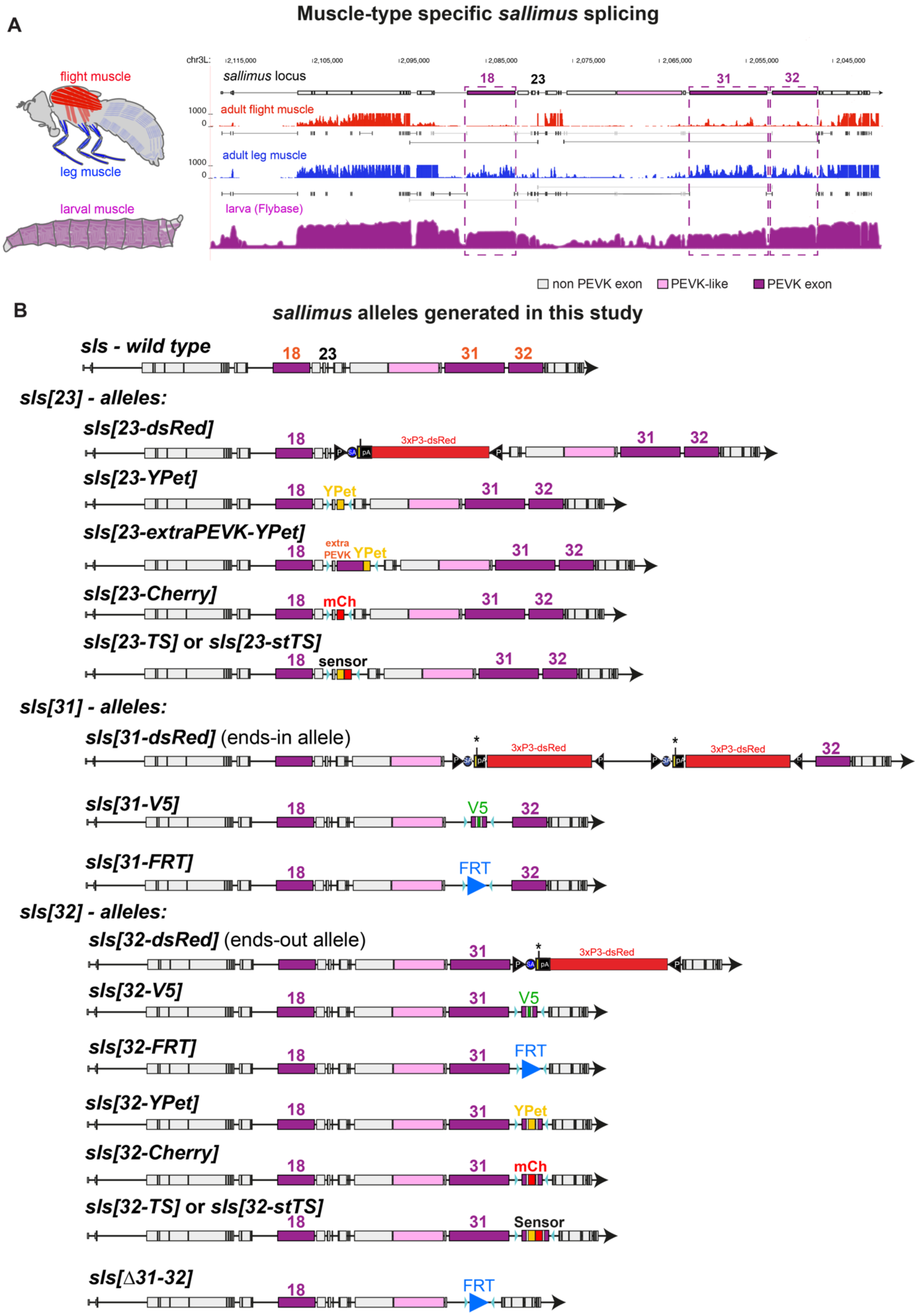
A
Muscle-type specific sallimus splicing
chr3L:
2,115,000
2,105,000
2,095,000
2,085,000
2,075,000
2,065,000
2,055,000
2,045,000
sallimus locus
18
23
31
32
flight muscle
leg muscle
larval muscle
1000
0
adult flight muscle
adult leg muscle
larva (Flybase)
non PEVK exon
PEVK-like
PEVK exon
B
sallimus alleles generated in this study
sls - wild type
18
23
31
32
sls[23] - alleles:
sls[23-dsRed]
3xP3-dsRed
sls[23-YPet]
YPet
sls[23-extraPEVK-YPet]
extra PEVK
YPet
sls[23-Cherry]
mCh
sls[23-TS] or sls[23-stTS]
sensor
sls[31] - alleles:
sls[31-dsRed] (ends-in allele)
3xP3-dsRed
3xP3-dsRed
sls[31-V5]
V5
sls[31-FRT]
FRT
sls[32] - alleles:
sls[32-dsRed] (ends-out allele)
3xP3-dsRed
sls[32-V5]
V5
sls[32-FRT]
FRT
sls[32-YPet]
YPet
sls[32-Cherry]
mCh
sls[32-TS] or sls[32-stTS]
Sensor
sls[Δ31-32]
FRT

Fig. S1 – *sallimus* (*sls*) alleles

(A) mRNA-SEQ read counts and junction reads (grey lines) in the *sallimus* locus from dissected adult *Drosophila* indirect flight muscles (red), adult legs (blue) and larvae (magenta). PEVK domains are highlighted in magenta. Data were extracted from (*36*) and Flybase. (B) Scheme of all new *sls* alleles generated in this study; PEVK regions are highlighted in magenta, the dsRed marker in red.

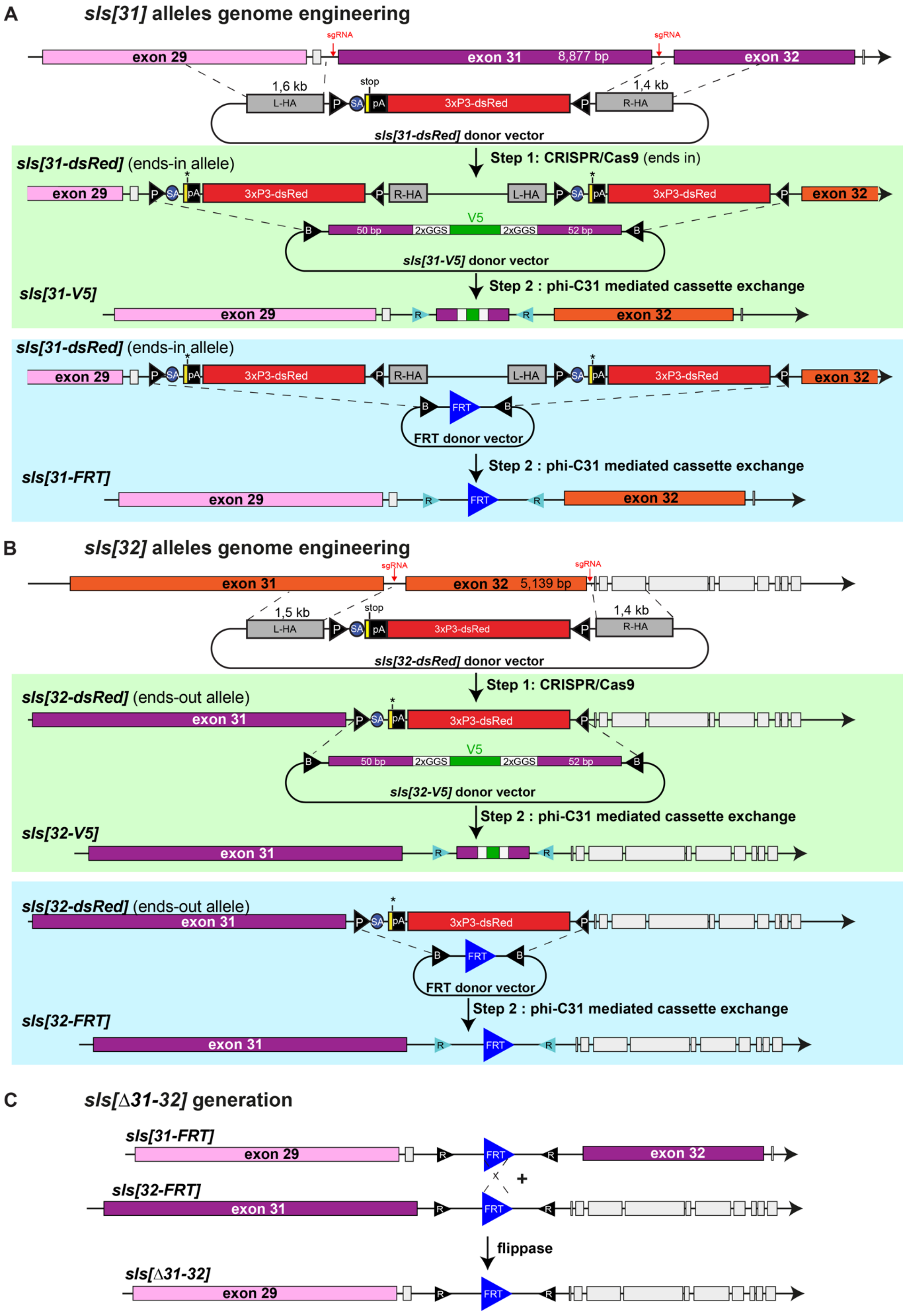
A
sls[31] alleles genome engineering
sgRNA
sgRNA
exon 29
exon 31
8,877 bp
exon 32
1,6 kb
stop
1,4 kb
L-HA
P
SA
pA
3xP3-dsRed
P
R-HA
sls[31-dsRed] donor vector
Step 1: CRISPR/Cas9 (ends in)
sls[31-dsRed] (ends-in allele)
R-HA
L-HA
V5
50 bp
2xGGS
2xGGS
52 bp
B
sls[31-V5] donor vector
Step 2 : phi-C31 mediated cassette exchange
sls[31-V5]
R
FRT
FRT donor vector
sls[31-FRT]
B
sls[32] alleles genome engineering
5,139 bp
1,5 kb
sls[32-dsRed] donor vector
Step 1: CRISPR/Cas9
sls[32-dsRed] (ends-out allele)
sls[32-V5] donor vector
sls[32-V5]
sls[32-FRT]
C
sls[Δ31-32] generation
+
flippase
sls[Δ31-32]

Fig. S2 – *sls[31]* and *sls[32]* CRISPR - RMCE editing

(A) Scheme showing the replacement or deletion of *sls* exon 31. Step 1: the target exon was replaced by a splice acceptor (SA)-3xstop-SV40 terminator (pA)-3xP3>dsRed cassette flanked by attP sites (P) using the CRISPR/Cas9 system (‘ends-in’ integration). Step 2: phi-C31-mediated cassette exchange (RMCE) was performed to replace the dsRed cassette with a V5 tag maintaining the splicing regulation of the exon 31 or with an FRT site. **(B)** The same strategy was used to replace or delete *sls* exon 32 (ends-out allele). **(C)** Generation of *sls[Δ31-32]* by flippase expression (under heat-shock control) in the germline of *sls[31-FRT] / sls[32-FRT]*. The successful deletion of both exons was identified by PCR.

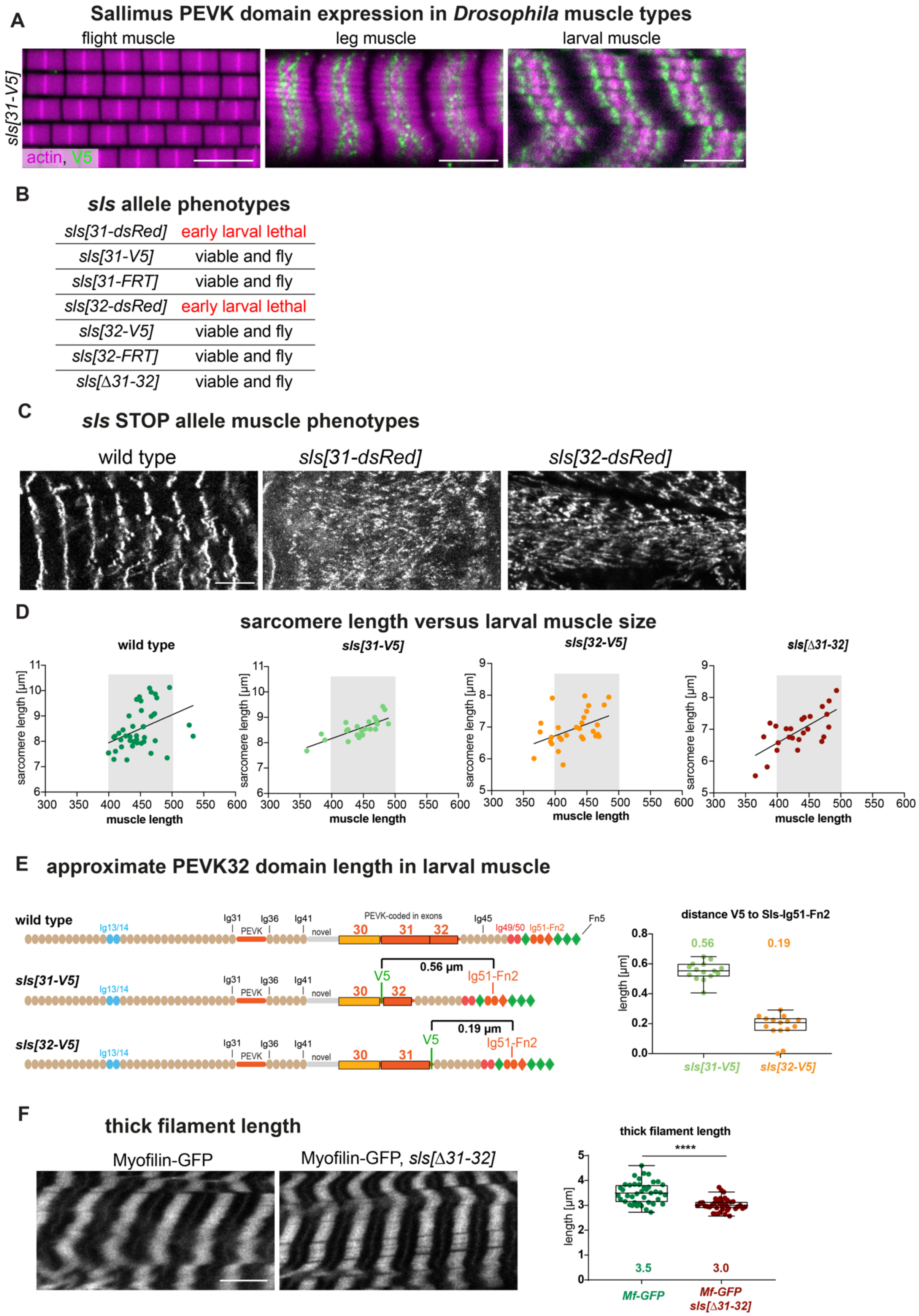

A
Sallimus PEVK domain expression in Drosophila muscle types
flight muscle
leg muscle
larval muscle
sls[31-V5]
actin, V5
B
sls allele phenotypes
sls[31-dsRed] early larval lethal
sls[31-V5] viable and fly
sls[31-FRT] viable and fly
sls[32-dsRed] early larval lethal
sls[32-V5] viable and fly
sls[32-FRT] viable and fly
sls[Δ31-32] viable and fly
C
sls STOP allele muscle phenotypes
wild type
sls[31-dsRed]
sls[32-dsRed]
D
sarcomere length versus larval muscle size
wild type
sls[31-V5]
sls[32-V5]
sls[Δ31-32]
sarcomere length [µm]
muscle length
E
approximate PEVK32 domain length in larval muscle
wild type
sls[31-V5]
sls[32-V5]
Ig13/14
Ig31
Ig36
Ig41
PEVK
novel
PEVK-coded in exons
30
31
32
Ig45
Ig49/50
Ig51-Fn2
Fn5
V5
0.56 µm
0.19 µm
distance V5 to Sls-Ig51-Fn2
length [µm]
0.56
0.19
F
thick filament length
Myofilin-GFP
Myofilin-GFP, sls[Δ31-32]
thick filament length
****
length [µm]
3.5
3.0
Mf-GFP
Mf-GFP sls[Δ31-32]

Fig. S3 – Sls-V5 expression and *sls* alleles sarcomere phenotypes

(A) Flight, leg and larval muscles of *sls[31-V5]* stained for actin (magenta) and V5 (green). Note the absence of V5 from flight muscles. Scale bars: 5 µm. **(B)** Viability of *sls* alleles. Note that *sls[31-dsRed]* and *sls[32-dsRed]* are lethal at early larval stages; the other *sls* deletion alleles are viable and can fly normally. **(C)** Stage 17 control wild-type embryo (*Zasp66-GFP / +; Df(3L)BSC366 / +*), compared to *sls[31-dsRed]* and *sls[32-dsRed]* embryos (*Zasp66-GFP / +; Df(3L)BSC366 / sls[31-dsRed]* and *Zasp66-GFP / +; Df(3L)BSC366 / sls[32-dsRed]*). Note the severely affected sarcomere pattern in the mutants. **(D)** Relation between VL3 muscle length and sarcomere length in wild type (*w[1118]*); *sls[31-V5]*; *sls[32-V5]* and *sls[Δ31-32]* third instar larvae. Muscles from 400 to 500 µm length (grey boxes) were used for quantifications in Fig. 2D. **(E)** Approximate length of Sls PEVK protein region encoded in *sls* exon 32, estimated by measuring the distance between V5 tag and Sls-Nano42 nanobody (recognizing Sls-Ig51-Fn2) in *sls[31-V5]* and *sls[32-V5]* (N=16). The difference between both indicates the PEVK32 length in larval sarcomeres. **(F)** Thick filament length by quantifying Mf-GFP signal length in wild-type *(Mf-GFP* N=41) and *sls[Δ31-32]* (N=38) VL3 larval muscles. Scale bar: 10 µm. Mann Whitney test, ****: $p < 0.0001$.

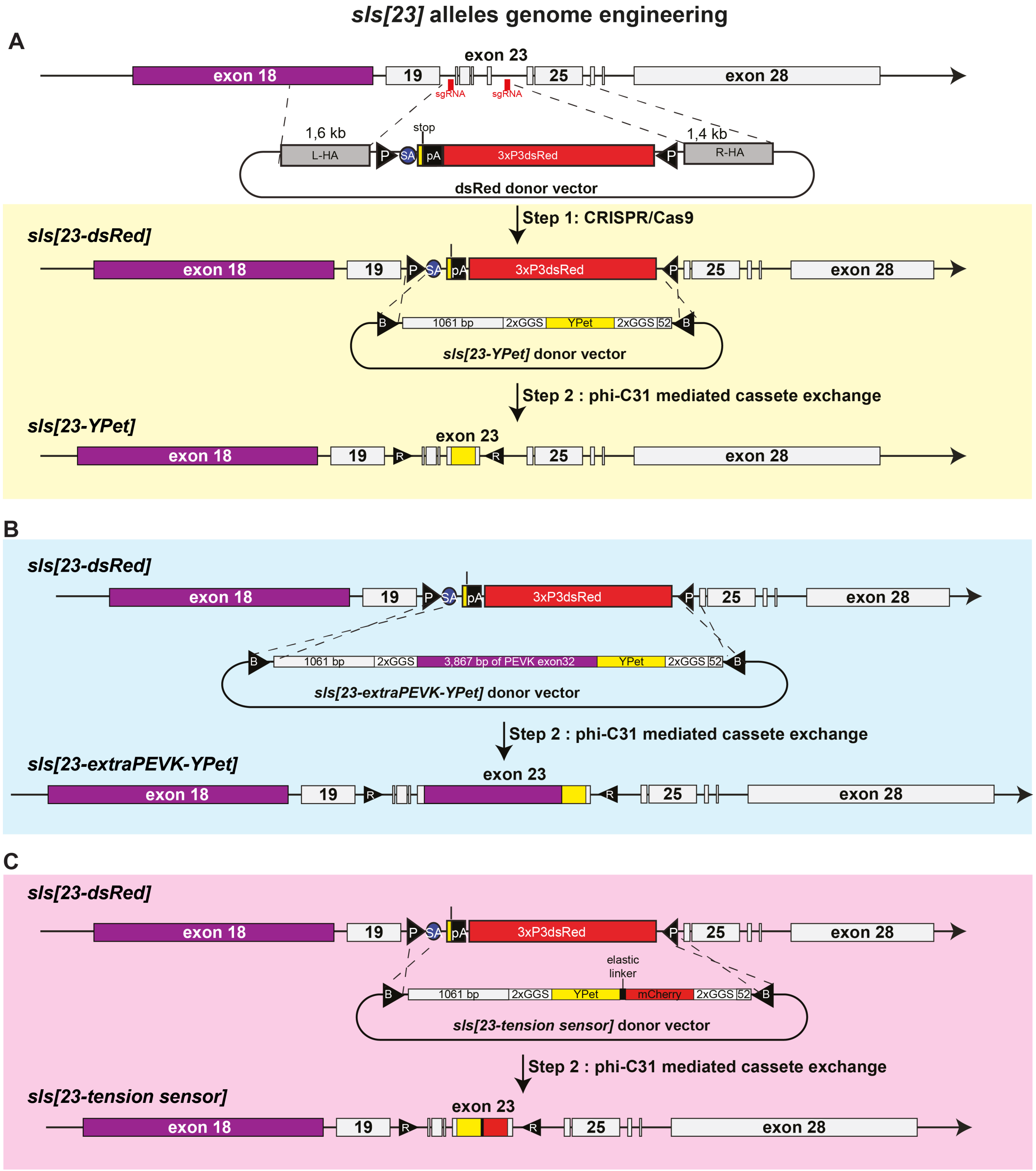

Fig. S4 – *sls[23]* CRISPR - RMCE editing

(A-C) Schemes showing the targeting of *sls* exon 23. **Step 1:** the *sls* exons 20 to 23 were replaced by a splice acceptor (SA)-3xstop-SV40 terminator (pA)-3xP3>dsRed cassette flanked by attP sites (P) using CRISPR/Cas9. **Step 2:** phi-C31-mediated cassette exchange was performed to replace the dsRed cassette with wild-type *sls* exons 20 to 22 and *sls* exon 23 with an inserted YPet (A, *sls[23-Ypet]*), or 3867 bp of *sls* exon 32 plus YPet (B, *sls[23-extraPEVK-Ypet]*) or different tension sensors (C).

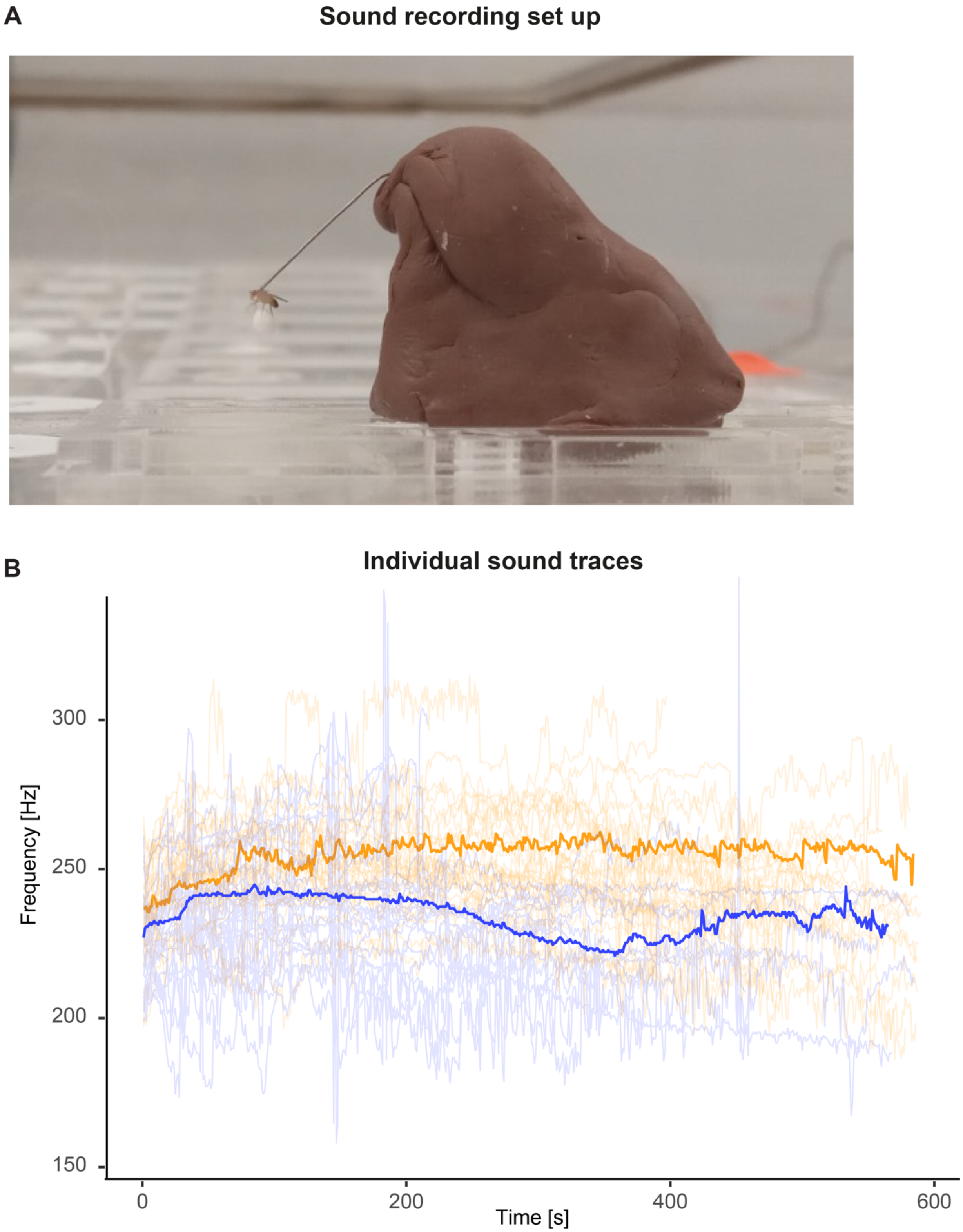

Fig. S5 – wing beat frequency set-up and recordings
(A) Wing beat sound recording set-up. The fly is tethered and rests on a paper ball. Upon removal of the ball, it starts to fly and the microphone below will record the sound frequency. (B) Individual sound traces recorded over 10 minutes. *sls[23-YPet]* plotted in orange and *sls[23-extraPEVK-*

YPet] in blue. One representative trace is highlighted. Note the lower frequency and the variations in *sls[23-extraPEVK-YPet]*.

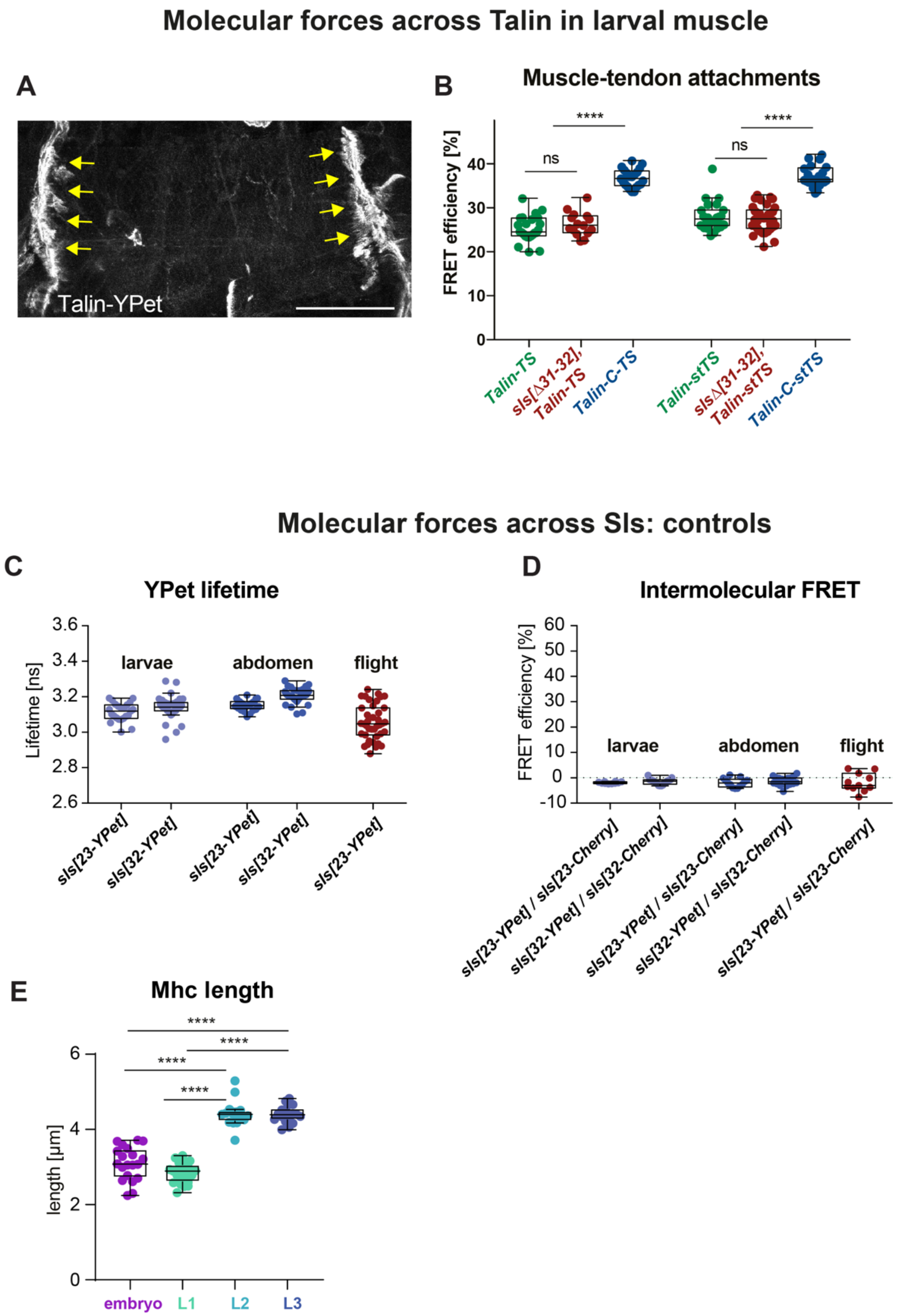

Fig. S6 – Talin and Sallimus molecular forces

(A) Expression of Talin (*rhea*)-YPet in larval muscle. Talin localises at the muscle attachments. **(B)** FLIM-based FRET quantification of Talin molecular forces comparing Talin-TS and Talin-stTS as well as C-terminal no force controls (Talin-C-TS, Talin-C-stTS) in wild type and *sls[Δ31-32]* living third instar larvae at muscle attachments. Note the reduction of FRET in Talin-TS and

Talin-stTS in wild type and *sls[Δ31-32]* compared to Talin-C-TS or Talin-C-stTS. Tukey's multiple comparisons test, ns: $p>0.05$, ****: $p<0.0001$, n between 15 and 34, see data S4 for details. **(C)** Fluorescence lifetime (FLIM) of *sls[23-YPet]* and *sls[32-YPet]* in larval, abdominal and flight muscles. n between 23 and 39, see data S4 for details**.** **(D)** Intermolecular FRET in larval, abdominal and flight muscles in the indicated trans-heterozygous genotypes. No intermolecular FRET is detected. n between 10 and 19, see data S4 for details. **(E)** Myosin length quantification of alive MhcGFP expressing embryos (N=20) and larvae at different larval instars (L1 N=24, L2 N=17, L3 N=18) used in Figure 4E. Tukey's multiple comparisons test, ****: $p<0.0001$.

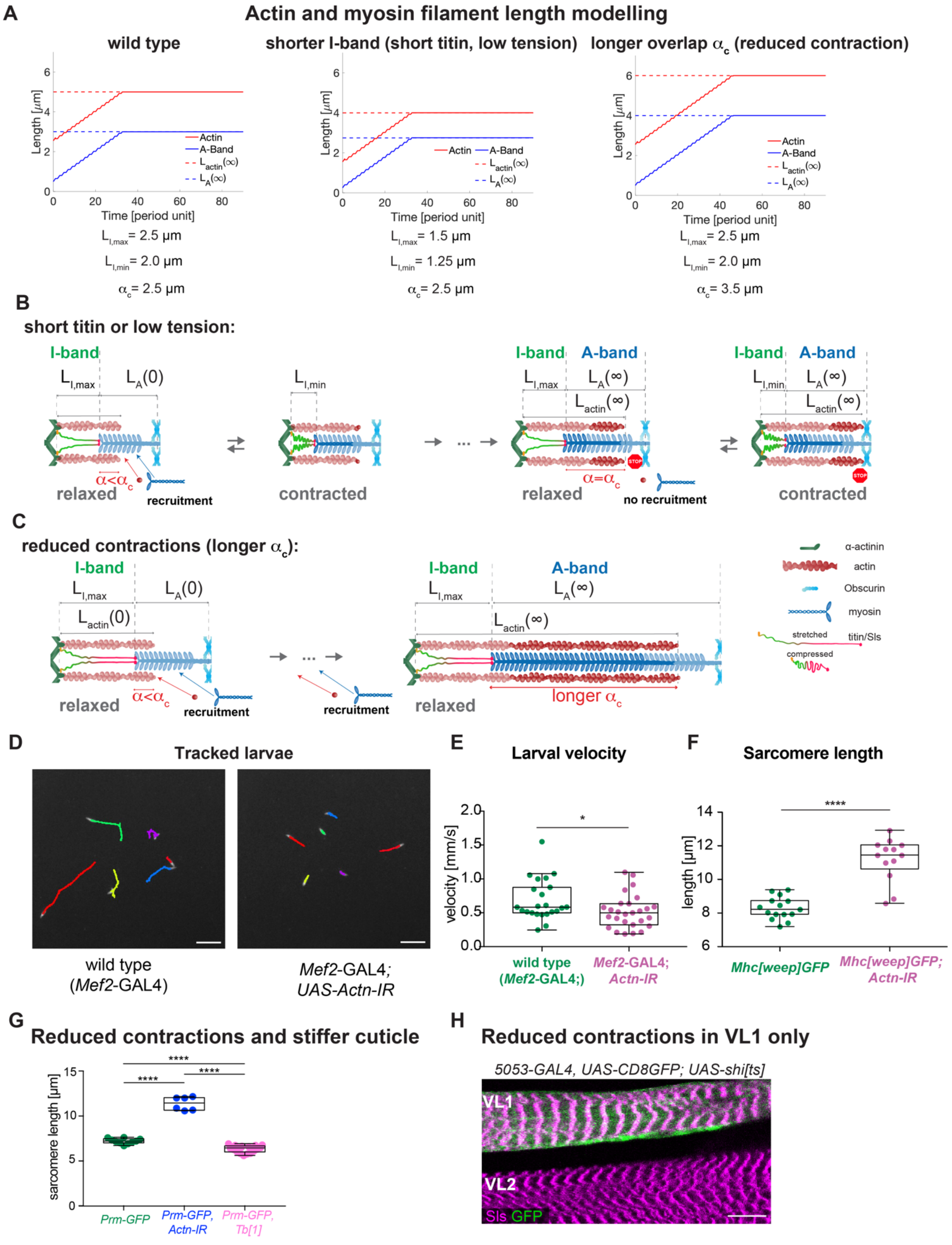

Fig. S7 – Actin-myosin filament length scaling

(A) Modelling of the actin and myosin filament length growth over time, as predicted by the mathematical model. Note a plateau that is approached for all three conditions. Left: wild type condition with long I-band resulting in a long A-band. Middle: short I-band mutant (or low tension) resulting in a short A-band. Right: increased actin-myosin overlap α (mimicking reduced contractions) resulting in longer I- and A-bands and longer actomyosin overlap. **(B)** Mathematical model scheme of a short PEVK *sls* mutant or low I-band tension, which corresponds to shorter I-band lengths (both in contracted and relaxed states). When $\alpha = \alpha_C$ is reached, no new subunits are recruited and only a shorter A-band length (compared to wild type in Figure 5A) is reached. **(C)** Mathematical model scheme of a sarcomere with reduced contraction (longer α_C) during development. Recruitment of actin and myosin subunits does not stop, hence a much longer myosin filament with a much longer actomyosin overlap (compared to wild type in Figure 5A) is generated. **(D)** Tracks of crawling larvae from Movie S1. Scale bar represents 1 cm. (**E**) Quantification of larval velocity of wild-type (N=23) and *Mef2*-GAL4, *Actn-IR* (N=27) larvae (Mann Whitney test, *: $p < 0.05$). **(F)** Sarcomere length quantification of wild type (*Mhc[weep]GFP, Mef2*-GAL4 N=15) and *Actinin* knockdown larvae (*Mhc[weep]GFP, Mef2*-GAL4, *Actn-IR* N=13). **(G)** Sarcomere length quantifications of wild type (*Prm-GFP, Mef2-GAL4* N=10), *Actinin* knockdown (*Prm-GFP, Mef2-GAL4, Actn-IR* N=6) or *Prm-GFP Tubby (Tb[1]* N=18). Tukey's multiple comparisons test, ****: $p<0.0001$. **(H)** Expression of *shibire^{TS}* only in VL1 muscle (marked by UAS-CD8-GFP in green) causes longer sarcomeres compared to neighbouring VL2 muscle (Z-disc labelled by Sls N-term in magenta). Scale bar is 20 µm.

Movie S1 (separate file) – Tracked wild type and *Mef2*-GAL4, *Actn-IR* larvae.

Table S1 (separate file) – Titin evolutionary tree, species and protein names.

Data S1 (separate file) – Archive of all titin protein FASTA sequences used for evolutionary tree in Figure 1.

Data S2 (separate file) – Data of Figure 2

Data S3 (separate file) – Data of Figure 3

Data S4 (separate file) – Data of Figure 4

Data S5 (separate file) – Data of Figure 5

File S1 (separate file) – Python script to calculate the PEVK content in the titin sequences.

File S2 (separate file) – Modelling code.
